# Exosomes from nicotine-stimulated macrophages accelerate atherosclerosis through miR-21-3p/PTEN-mediated VSMC migration and proliferation

**DOI:** 10.7150/thno.37357

**Published:** 2019-09-21

**Authors:** Jumo Zhu, Bei Liu, Zhiyan Wang, Di Wang, Huaner Ni, Lili Zhang, Yi Wang

**Affiliations:** 1 Department of Cardiology, Shanghai General Hospital, School of Medicine, Shanghai Jiao Tong University, Shanghai, China.; 2 Department of Cardiology, Ruijin Hospital, School of Medicine, Shanghai Jiao Tong University, Shanghai, China.; 3 Department of Cardiology, Shanghai Jiao Tong University Affiliated Sixth People's Hospital, Shanghai, China.

**Keywords:** Exosomes, Macrophage, Nicotine, Vascular smooth muscle cell, Atherosclerosis, miR-21-3p, PTEN

## Abstract

**Rationale:** During the development of atherosclerosis, macrophages secrete exosomes that regulate vascular smooth muscle cells (VSMCs); however, whether nicotine, a major constituent of cigarettes, can modulate this communication in the context of atherogenesis remains to be further studied. In this study, we hypothesized that nicotine induces macrophages to secrete atherogenic exosomes containing microRNAs (miRNAs) to mediate cell-to-cell crosstalk and encourage proatherogenic phenotypes of VSMCs.

**Methods:** In an *in vivo* study, nicotine was administered subcutaneously to 8-week-old male *ApoE^-/-^* mice fed a high-fat diet (HFD) for 12 weeks. Oil red O and hematoxylin and eosin (HE) were used to stain atherosclerotic lesions. Lesion macrophages, VSMCs and exosomes were stained for CD68, α-smooth muscle actin (α-SMA) and CD9, and plaque exosomes were observed by transmission electron microscopy (TEM). Exosomes derived from control macrophages (M-Exos) and from nicotine-treated macrophages (NM-Exos) were isolated by ultracentrifugation, purified by sucrose density gradient centrifugation and characterized based on specific morphology and surface markers. The IVIS® Spectrum *in vivo* imaging system showed the biodistribution of NM-Exos and M-Exos in circulation. Chitosan hydrogel-incorporated exosomes were applied to simulate exosome secretion *in situ*. Scratch wound assay, transwell assay and EdU staining were conducted to assess the effects of NM-Exos on the migration and proliferation of mouse VSMCs. RNA-seq was performed to determine the miRNA profiles of M-Exos and NM-Exos. Quantitative real-time PCR (qRT-PCR) analysis was conducted to detect the expression levels of miRNAs and mRNAs. The roles of the candidate miRNA and its target gene were assessed using specific RNA inhibitors, siRNAs and miRNA mimics. Western blotting was used to detect candidate protein expression levels. A dual-luciferase reporting system was utilized to confirm the binding of a specific miRNA to its target gene.

**Results:** Nicotine induced atherosclerotic lesion progression and resulted in plaque exosome retention *in vivo*. The biodistribution of NM-Exos showed that plaque-resident exosomes might be secreted *in situ*. VSMCs cocultured *in vitro* with nicotine-stimulated macrophages presented an increased capacity for migration and proliferation, which was exosome-dependent. In addition, isolated NM-Exos helped promote VSMC migration and proliferation. miRNA profiling showed that miR-21-3p was enriched in NM-Exos, and this miRNA was shown to play a key role in regulating NM-Exos-induced effects by directly targeting phosphatase and tension homologue (PTEN).

**Conclusion:** Exosomal miR-21-3p from nicotine-treated macrophages may accelerate the development of atherosclerosis by increasing VSMC migration and proliferation through its target PTEN.

## Introduction

Cardiovascular disease, one of the leading causes of human death worldwide, is primarily attributed to atherosclerosis 1. Generally, atherosclerosis is a lipid-driven progressive inflammatory disease in which excess cholesterol deposition in the atrial wall results in a nonresolving immune response 2. In the initial stages, atherosclerotic lesions, termed fatty streaks, consist of lipid-laden macrophages called foam cells. These early lesions are followed by fibrous lesions, which are characterized by the formation of necrotic debris and the presence of VSMCs 3.

Crosstalk between macrophages and VSMCs is essential in regulating atherosclerosis progression. While these two cells are classically described to communicate via the secretion of soluble factors such as cytokines and growth factors, alternative mechanism have recently been studied 4-6. One such mechanism involves small membranous particles secreted from macrophages, termed exosomes, that contribute significantly to the intercellular communication and subsequent function reprogramming of the VSMCs 7. Exosomes are nanosized (30 to 120 nm) vesicles of endocytic origin that carry biological content from donor to target cells under both physiological and pathological conditions 8. Accumulating evidence suggests that lesion macrophages transfer exosomal miRNAs, thereby coordinating gene expression in recipient cells and influencing plaque development 9.

Exposure to cigarette smoke is a known risk factor of atherosclerosis 10-12. Nicotine, the major component of cigarette smoke, not only directly activates the plaque cell migration and proliferation but also intensifies the proinflammatory communication of cytokines between macrophages and VSMCs, subsequently prompting atherogenesis 13-15. Nevertheless, it is unclear whether nicotine exposure augments atherosclerosis by inducing the secretion of macrophage-derived exosomes in VSMCs.

In this study, we investigated the potential role of exosomes from nicotine-treated macrophages (NM-Exos) in VSMCs. Our research shows that VSMCs are regulated by exosomal miR-21-3p secreted by macrophages exposed to nicotine, which targets PTEN and facilitates atherogenesis.

## Methods and Materials

### Animal model

Eight-week-old male *ApoE^-/-^* mice (C57BL/6) from Cavens Lab Animal (Changzhou, China) were used to generate an atherosclerosis model. Those mice were randomized into 3 groups: normal chow diet (NCD, TD08485, Harlan Teklad) (n=10), high-fat diet (HFD, TD02028, Harlan Teklad) (n=10), and nicotine + HFD (n=10). To monitor and analyze the influence of nicotine, mice in the nicotine+ HFD group were treated with 2 mg·kg^-1^·day^-1^ of nicotine via subcutaneous injection, while those in the NCD and HFD groups were injected with an equal volume of phosphate buffer saline (PBS). The treatment lasted for 12 weeks. All mice were ultimately euthanized for further analysis. All animal experiments were previously approved by the Animal Care and Use Committee at the Research Institute of Medicine of Shanghai Jiao Tong University.

### Cell culture

A line of mouse vascular smooth muscle cells (ATCC, USA) was cultured in high-glucose Dulbecco's modified Eagle medium (Gibco BRL, Grand Island, USA) with 10% foetal bovine serum (Gibco BRL). The mouse macrophage cell line RAW264.7 (ATCC) was also cultured in high-glucose Dulbecco's modified Eagle medium (Gibco BRL) with 10% foetal bovine serum (Gibco BRL). Cells were maintained at 37°C with 5% CO_2_ in a humidified environment.

### Immunofluorescence staining

Briefly, plaque samples at week 12 were fixed in 4% paraformaldehyde, dehydrated in a 30% sucrose solution and embedded in optimal cutting temperature compound (OCT) (Sakura Finetek, USA, 4583). Four-micron-thick sections were incubated with primary antibody: CD68 (Abcam, Cambridge, Britain, ab53444, 1:100), α-SMA (Abcam, ab7817, 1:100) and CD9 (Abcam, ab92726, 1:100) overnight at 4 °C, followed by secondary antibody at room temperature for 1 h away from light. Images were observed with a fluorescence microscope (Leica DMI6000B, Germany).

### Immunohistochemical staining

Four-micron-thick plaque sample sections were incubated with the α-SMA antibody (Abcam, ab7817, 1:100) and the PTEN antibody (CST, 9188, 1:100) overnight at 4 °C after blocking with 1% goat serum. Slides underwent colour development with DAB and haematoxylin counterstaining. Images were obtained with an optical microscope (Leica DMI6000B).

### Exosome isolation

RAW264.7 macrophages at 70-80% confluence were cultured in high-glucose Dulbecco's modified Eagle medium (Gibco BRL) with 10% exosome-depleted foetal bovine serum with or without nicotine (Sigma-Aldrich, USA, 10^-5^ M) for 48 h. The methods were previously described 16, 17. Briefly, the culture medium was transferred to 50 mL centrifuge tubes and centrifuged at 300×g for 10 min to collect the supernatant. Subsequently, the supernatant underwent a series of low-speed centrifugation steps (2,000×g for 10 min) to discard cell debris. Then, the supernatant was centrifuged at 10,000×g for 30 min followed by ultracentrifugation for 120 min at 100,000×g (Optima L-100XP, Beckman Coulter, USA). The pelleted exosomes were washed twice with a large volume of PBS and centrifuged at 100,000×g for 120 min.

### Sucrose density gradient centrifugation

Sucrose density gradient fractionation was conducted for exosome purification. Isolated exosomes were resuspended in 0.25 M sucrose and loaded onto a step gradient with layers of 2, 1.3, 1.16, 0.8, and 0.5 M sucrose followed by ultracentrifugation at 100000×g for 2.5 h 18, 19. Subfractions containing different densities were sequentially extracted from the top of the sample tube and analysed by SDS-PAGE with exosome marker proteins. Then, the suspension containing exosomes was washed twice with a large volume of PBS and centrifuged at 100,000×g for 120 min. All procedures were performed at 4 °C. The concentrated exosomes were stored at -80 °C or used for the subsequent experiments.

### Exosome protein quantitation assay

A MicroBCA Protein Assay Kit (Thermo Fisher Scientific, USA, 23235) was used to determine the total protein content of exosomes from control macrophages (M-Exos) and NM-Exos collected from 10^6 cells over 48 h. M-Exos and NM-Exos were lysed using RIPA buffer (Beyotime, China) to prepare protein samples of unknown concentration. Albumin provided in the kit was used to prepare standard samples of eight known concentrations. The standard and unknown samples were added to 96-well plates and mixed with the working reagent. The mixture was incubated at 60 °C for 60 min, and the absorbance was measured at 562 nm on a plate reader. A standard curve was prepared by plotting the average 562 nm reading for each albumin standard versus its concentration and then used to determine the protein concentration of each unknown sample. Finally, total protein in M-Exos and NM-Exos lysates was calculated.

### TEM

For tissue TEM observation, mouse aortas were fixed with 2.5% glutaraldehyde and postfixed with 3% osmium tetroxide (OsO4) for 2 h. The specimen was dehydrated in a graded series of ethanol, embedded in Epon resin and then sliced into 100 nm pieces and finally imaged with a transmission electron microscope at 80 kV (Hitachi, Japan, H7500 TEM).

For cell TEM observation, the VSMCs were cocultured with exosomes for 120 min and then fixed with 2.5% glutaraldehyde and postfixed with 3% osmium tetroxide (OsO4) for 2 h. The specimen was dehydrated in a graded series of ethanol, embedded in Epon resin and then imaged with a transmission electron microscope at 80 kV (H7500 TEM).

For exosome TEM observation, exosomes were fixed with 2.5% glutaraldehyde at 4 °C overnight. After washing, vesicles were loaded onto formvarcarbon-coated grids, negatively stained with aqueous phosphotungstic acid for 60 s and imaged with a transmission electron microscope at 80 kV (H7500 TEM).

### Size distribution and particle concentration

The size of the vesicles was determined by a dynamic light scattering (DLS) technique using a ZetasizerNano ZS90 analysis system (Malvern Instruments, UK, Zetasizer version 7.12). A size distribution plot, for number particle size distribution (PSD), with the x-axis showing the distribution of estimated particle diameter (nm) and the y-axis showing the relative percentage, was created.

Nanoparticle tracing analysis (NTA) was performed by Nanosight (Merkel Technologies Ltd., Israel, NTA version NTA 3.2 Dev Build 3.2.16) to characterize the concentration of M-Exos and NM-Exos. M-Exos and NM-Exos collected from 10^7^ cells over 48 h were diluted in 1 ml of PBS and evaluated. A particle size distribution (PSD) plot was created with estimated particle diameter (nm) on the x-axis and concentration (particles/mL) on the y-axis. Additionally, a PSD plot based on intensity was generated with estimated particle diameter (nm) on the x-axis and intensity (a. u.) on the y-axis.

### Exosome labelling and cellular uptake

Purified exosomes were labelled with the membrane-labelling dye PKH67 (Invitrogen, USA) according to the manufacturer's instructions and were then washed and resuspended in serum-free medium. Next, VSMCs were seeded into glass bottom dishes (Cellvis, Mountain View, CA, US) for single layer and then cocultured with PKH67-labelled vesicles for 30 min, 60 min and 120 min; washed with PBS three times; fixed in 4% paraformaldehyde; stained with DiI (Invitrogen); washed with PBS; and imaged by confocal microscopy (Leica TCS SP8).

### *In vivo* imaging of fluorescently labelled exosomes and tracking

PKH67-labelled exosomes derived from nicotine-induced macrophages and control macrophages were injected intravenously either into *ApoE^-/-^* mice (20 mg of exosomes/mouse). At 21 days after injection, various tissues were harvested (lung, spleen, kidney, liver, and heart) for *in vivo* and *ex vivo* imaging. The intensity of fluorescence was quantified using the IVIS® Spectrum system and Living Image Software (PerkinElmer) to assess tissue distribution of PKH67-labelled exosomes 20, 21.

### Preparation of chitosan hydrogel and simulation of exosomes secretion *in situ*

The methods were described previously 22, 23. Briefly, a stock solution of 2% chitosan (Mn =50,000) was prepared by dissolving the chitosan powder (Sangon, China) in 0.1 M acetic acid, sterilized by filtering, and stored in an ice bath. The 50% β-glycerophosphate (β-GP) solution was prepared in tridistilled water, followed by sterilization with 0.2 μm filters. The β-GP solution was added into the chitosan solution in a 5:1 volume ratio under stirring inside the ice bath. Finally, the two solutions were fully mixed. To get the chitosan hydrogel-incorporated exosomes, 100 μg of exosomes was mixed with isovolumetric 2% chitosan hydrogel, ultimately getting the working solution. The final concentration of chitosan was 1%. After incubating at 37 °C for 30 min, the exosomes solution could cross-link into the hydrogel. Besides, exosomes were stained with DiI (Invitrogen). The chitosan hydrogel-incorporated exosomes were applied surrounding the *ApoE^-/-^* mice abdominal aorta by surgery and the aorta were checked at the 3 rd week.

### Coculture

A transwell (Corning, USA, 3412, 0.4 µm) coculture system was used as shown in Figure 5A. VSMCs alone, nicotine-treated VSMCs and VSMCs cocultured with untreated macrophages were used as controls. Nicotine (10^-5^ M) was added for macrophage stimulation. GW4869 (Sigma-Aldrich, USA, 10 μM) was added to block exosome secretion. Cells were cocultured for 24 h, and VSMC migration and proliferation assays were subsequently performed.

### miRNA expression profiling

Total RNA was isolated using TRIzol reagent (Takara Biotechnology, Japan) according to the manufacturer's instructions. Total RNA was separated on a 15% Tris-borate-EDTA (TBE) polyacrylamide gel (Invitrogen), and a band corresponding to small RNAs (18-30 nt) was excised. Isolated small RNAs were terminally repaired by adding 5′- and 3′-adaptors, reverse transcribed into cDNA and amplified. Raw small RNA sequence data were obtained using an Illumina HiSeq™ 2000 machine. After sequencing, Solexa CHASTITY quality-filtered reads were harvested as clean reads. The trimmed reads were aligned to miRBase v21 using NovoAlign software (v2.07.11), allowing a maximum of one mismatch. miRNAs in M-Exos and NM-Exos were profiled in two biological replicates. We searched for differences in each miRNA between the M-Exos and NM-Exos samples with a paired two-sided *t-test* and corrected for multiple testing using the Bonferroni method. Counts per million (CPM) were used for calculations. The fold change (mean CPM of each miRNA in the NM-Exos group / mean CPM of each miRNA in the M-Exos group) and P-values were calculated for each miRNA. These P-values were used to calculate the false discovery rate (FDR) for each miRNA, which was further used as a filter to identify significant miRNAs with a fold change ≥2 or ≤0.05 and an FDR<0.05. We analyzed expression data with Multiple Experiment Viewer (MEV) software. To visualize miRNA expression data, CPM values were normalized by Z-score transformation 20, 24 as shown below:


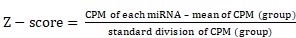


TargetScan (http://www.targetscan.org/), miRWalk (http://mirwalk.umm.uni-heidelberg.de) and miRbase (http://www.mirbase.org/) were applied to predict the target genes of miRNAs, respectively. Common genes predicted different algorithms were retained as target genes.

### qRT-PCR analysis

Total RNA was extracted from cultured cells using TRIzol Reagent (Takara Biotechnology). For mRNA detection, cDNA was synthesized from 1 μg of total RNA by using a PrimeScript™ RT Master Mix (Takara Biotechnology, Japan). Then, qRT-PCR analysis was performed with SYBR Premix Ex TaqTM (Takara Biotechnology, Japan). The relative standard curve method (2-△△CT) was used to determine the relative mRNA expression using GAPDH as follows: mmu-PTEN: forward, 5'-TTGGCGGTGTCATAATGTCT-3' and reverse, 5'-GCAGAAAGACTTGAAGGCGTA-3'; and mmu-GAPDH: forward, 5'-GTGGCAAAGTGGAGATTGTTGCC-3' and reverse, 5'-GATGATGACCCGTTTGGCTCC-3'. For miRNA analysis, exosomal miRNAs were isolated by using the SeraMir Exosome RNA Purification Kit (System Biosciences, Mountain View, USA), and cDNA for miRNAs was synthesized using the TaqMan microRNA assay kit (Applied Biosystems, Foster City, USA) as described in the manufacturer's protocol. The qRT-PCR reaction was performed using FastStart Universal SYBR Green Master Mix (Roche, Indianapolis, USA) with the miRNA-specific forward primer and the universal reverse primer (RiboBio, Guangzhou, China). U6 small nuclear RNA was used to normalize the results.

### RNA interference

miR-21-3p inhibitors (RiboBio, miR20004628) and their negative control inhibitors were purchased from RiboBio. Cell transfection was performed following the handbook from RiboBio. Briefly, the cells were cultured in 6-well culture plates and transfected with miR-21-3p inhibitors or the negative control inhibitors using Lipofectamine 3000 (Invitrogen) and cultured in exosome-depleted medium with 80 μg/mL NM-Exos (160 μg/well) or an equal volume of PBS. After 24 h of incubation, the subsequent experiments were performed.

miR-21-3p mimics (RiboBio, miR10004628) together with negative control mimics and Cy3-labelled miR-21-3p mimics were purchased from RiboBio. Cell transfection was performed following the handbook from RiboBio. Briefly, the cells were cultured in 6-well culture plates and transfected with miR-21-3p mimics or the negative control mimics using Lipofectamine 3000 (Invitrogen). After 24 h of incubation, the subsequent experiments were performed.

PTEN siRNAs (siPTEN#1, siPTEN#2 and siPTEN#3) obtained from RiboBio were used to knockdown the expression of PTEN in VSMCs. siPTEN#2 was selected according to the western blotting results (Figure. S2F). Briefly, cells were transfected with siPTEN or the universal negative control siRNA using Lipofectamine 3000 (Invitrogen) according to the manufacturer's instructions. Thirty-six hours later, the inhibitory efficiency of these siRNAs was verified by western blotting, and the cells were used for the subsequent functional experiments. The same experiments were performed on cells treated with NM-Exos (80 μg/mL) or an equal volume of PBS.

### Migration assay

For the scratch wound assay, 3 × 10^5^ cells/well (three replicates per group) were plated into a 12-well plate and grown to confluence. The monolayer was scratched using a pipette tip and washed with serum-free medium to remove detached cells. Then, the cells were cultured in exosome-depleted medium supplemented with or without NM-Exos (80 μg/well) and miR-21-3p inhibitors; M-Exos were used as a control. VSMCs were photographed at 0 h, 12 h and 24 h after wounding. Wound closure was calculated as follows: migration area (%) = (A_0_ - A_n_)/A_0_ × 100, where A_0_ represents the initial wound area, and A_n_ represents the wound area at the time of measurement.

For the transwell assay, 1 × 10^4^ cells/well (three replicates per group) were suspended in low-serum (2% foetal bovine serum) medium and seeded into the upper chamber of 24-well transwells (Corning, USA, 3422) with 8 μm pore filters. Then, exosome-depleted medium with or without NM-Exos (80 μg/well) and miR-21-3p inhibitors was placed in the lower chamber; M-Exos were used as a control. After 24 h, the cells attached to the upper surface of the filter membranes were removed, and migrated cells on the lower surface were stained with 0.5% crystal violet for several minutes. Cell migration was observed under an optical microscope (Leica DMI6000B).

### EdU staining

The effects of NM-Exos on cell proliferation were determined by a cell counting EdU staining (Riobio, Guangzhou, China). Briefly, 2 × 10^3^ cells/well (four replicates per group) were seeded into 96-well plates and cultured in medium supplemented with or without NM-Exos (8 μg/well) and miR-21-3p inhibitors; M-Exos were used as a control. Then, 10 nM EdU was added for 12 h. The wells were washed with PBS, and the cells were fixed in 4% paraformaldehyde and stained according to the manufacturer's instructions. Cell proliferation was observed under a fluorescence microscope (Leica DMI6000B).

### Western blotting

The samples were lysed using RIPA buffer (Beyotime, China). Protein extracts were separated by sodium dodecyl sulfate-polyacrylamide gel electrophoresis (SDS-PAGE) and transferred to polyvinylidene fluoride membranes (Immobilon P, Millipore, USA). Blots were blocked with 5% milk in Tris-buffered saline containing 0.1% Tween-20 for 1 h at room temperature. The membranes were incubated with primary antibodies at 4 °C overnight, followed by incubation with horseradish peroxidase-conjugated secondary antibodies at 37 °C for 1 h. The antibodies included anti-PTEN (Abcam, ab32199, 1:1000), anti-CD9 (Abcam, ab92726, 1:1000), anti-TSG101 (Abcam, ab125011, 1:1000), anti-Annexin II (Abcam, ab178677, 1:1000), anti-calnexin (Abcam, ab92573, 1:1000), anti-GAPDH (Abcam, ab9485, 1:1000), caspase 3 (Proteintech, 19677-1-AP, 1:1000) and horseradish peroxidase-conjugated goat anti-rabbit IgG (Abcam, ab205718, 1:5000). The immunoreactive bands were visualized using an enhanced chemiluminescence reagent (Thermo Fisher Scientific, USA) and imaged by the ChemiDoc XRS Plus luminescent image analyser (Bio-Rad, USA). Densitometric quantification of band intensity from four independent experiments was carried out with Image-Pro Plus 6.0 software, and the relative expression level of the target protein was normalized to the band intensity of GAPDH.

### Dual luciferase assay

A total of 862 bp of the mmu-PTEN 3′UTR region was amplified and cloned into a dual-luciferase reporter vector (Promega, USA), referred to as psiCHECK2-PTEN-WT. A mutant type of the mmu-PTEN 3′UTR reporter plasmid (psiCHECK2-PTEN-MUT) was also generated by mutating the seed region for miR-21-3p. To evaluate the direct binding between miR-21-3p and the 3′UTR of PTEN, HEK-293 cells were co-transfected with 100 nM miRNA mimics and 1 μg of dual-luciferase reporter vector (psiCHECK2-PTEN-WT or psiCHECK2-PTEN- MUT). Luciferase activity was measured using the Dual-Glo luciferase assay system (Promega) 48 h after transfection. Normalized firefly luciferase activity was compared between groups.

### Statistical analysis

The data are expressed as the means ± standard deviation (SD). *Student's t test* (for comparisons between two groups) and one‐way *NOVA* (for comparisons among three or more groups), followed by the Bonferroni post‐hoc test were used for the statistical analysis. The statistical analysis was conducted using the SPSS 21.0 software. The values of P < 0.05 were considered significant.

## Results

### Nicotine increases lesion VSMCs and promotes exosome retention

**Figure 1:**
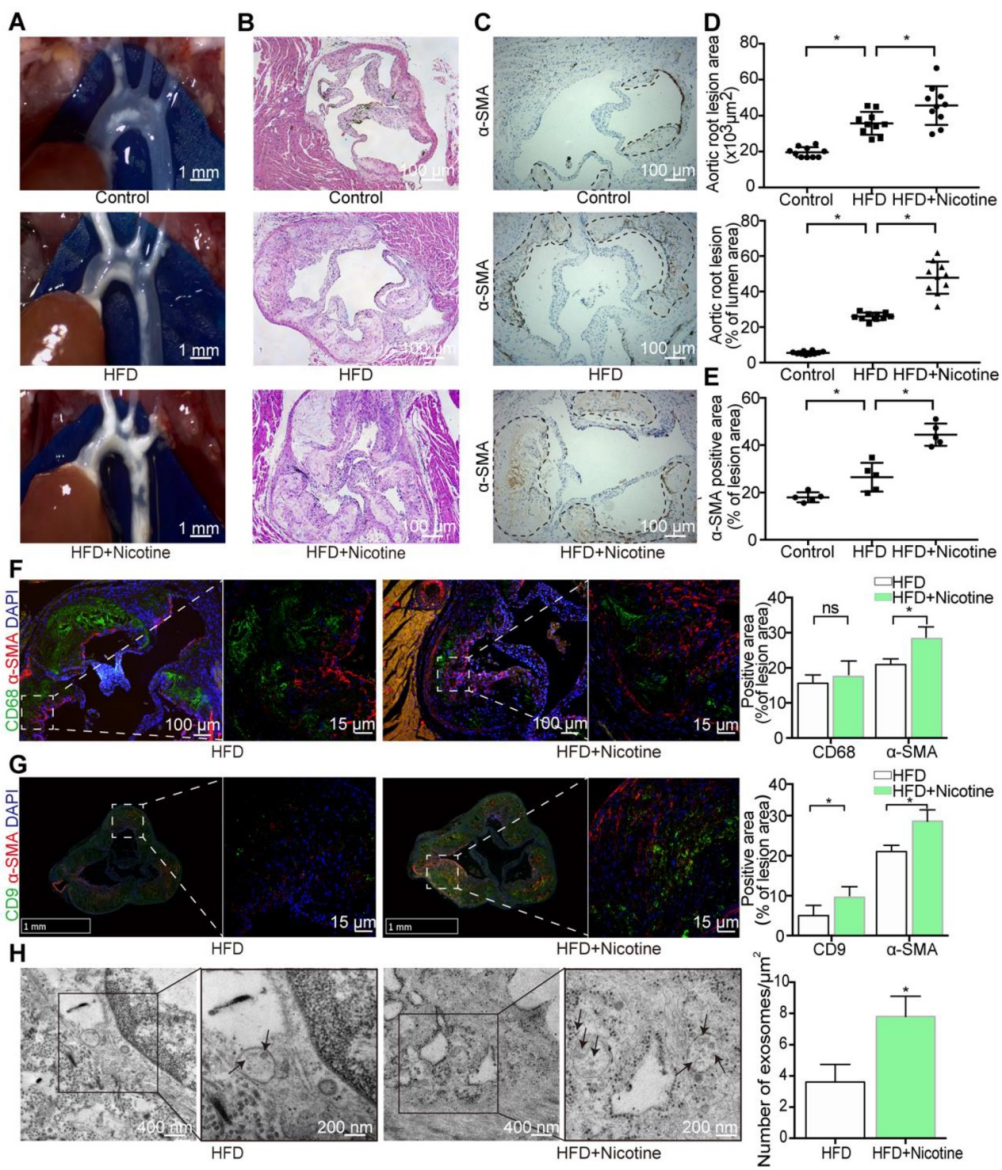
** Nicotine promotes atherosclerosis in mice.** (A) *ApoE^-/-^* mouse thoracic aorta of control, HFD and HFD+Nicotine group. Magnification ×1. (B) Aortic root atherosclerotic lesion of control, HFD and HFD+Nicotine group mice (n=10) were stained for hematoxylin and eosin (HE). Magnification ×100. (C) Immunohistochemical images stained for α-SMA of aortic root atherosclerotic lesion (n=5). Magnification ×100. (D) Quantification of aortic root lesion size and percentage of lumen area (n=5) for (B). (E) Quantification of α-SMA expression (n=5) for (C). (F) Immunofluorescent images stained for CD68 and α-SMA and quantification of expression(n=5). Magnification ×100 and ×630. (G) Immunofluorescent images stained for CD9 and α-SMA and quantification of expression (n=5). Magnification ×15 and ×630. (H) TEM images of plaque exosomes in each group and quantification (n=5). Magnification ×11000 and × 23000. *P<0.05; ns, not significant. All the data are presented as mean±SD (One-way *NOVA* and* Student's t-test*).

Nicotine was administered to *ApoE^-/-^* mice with atherosclerosis to determine whether nicotine stimulation increases susceptibility to atherosclerosis. Initial experiments confirmed the concurrent generation of mouse models of nicotine-induced and HFD-induced atherosclerosis (Figures 1A and S1A). Compared with the HFD mice, the nicotine-treated HFD-fed mice (HFD+nicotine) exhibited a significantly larger atherosclerotic lesion area in the aortic arches (0.36±0.02 mm^2^ vs. 0.46±0.03 mm^2^, P=0.0227) and an increased lesion area ratio (26±0.66% vs. 48±2.85%, P<0.05) (Figure 1B and 1D). Furthermore, nicotine administration changed the serum levels of glucose, total cholesterol, low-density lipoprotein cholesterol, high-density lipoprotein cholesterol, triglycerides, and uric acid, as well as systolic and diastolic blood pressure, but the differences were not statistically significant compared to HFD alone (Table S1). In short, the data indicated that nicotine promotes the progression of atherosclerosis.

The development of atherosclerosis involves the infiltration of macrophages from the lumen and the migration of VSMCs from the media into the intima 25. To detect changes in these cell components in nicotine-induced atherosclerotic lesions, aortic arches from the HFD+nicotine group were stained with CD68 (macrophages) and α-SMA (VSMCs) and analyzed by immunofluorescence (Figure 1F). We found that lesion macrophage content increased slightly but not significantly in the HFD+nicotine group (15.6±2.4% vs. 18.0±3.9%, P=0.273) (Figure 1F). However, the population of α-SMA-positive VSMCs increased greatly (21.1±1.58% vs. 28.6±2.88%, P=0.001) (Figure 1F). In addition, α-SMA expression was validated by immunohistochemical analysis (Figure 1C and 1E). The findings emphasized the key role of VSMCs in this nicotine stimulation model.

Many studies have shown that in the context of atherogenesis, in addition to the cells present, a number of exosomes are secreted throughout the plaques, and these exosomes have recently emerged as participants in the development of atherosclerosis 26-30. However, it was still uncertain whether this phenomenon also occurs in our nicotine stimulation model. To compare exosome quantity in plaques, sections were stained with CD9, an exosome marker. The results showed a much stronger CD9 signal in the plaque area in the nicotine group, implying greater exosome retention (9.1±2.72% vs 4.7±2.44%, P=0.0131) (Figure 1G). Additionally, the plaque exosomes were quantified directly by TEM, which showed obvious accumulation in the nicotine group (3.79±1.85 vs. 7.84±2.41, P<0.05) (Figure 1H). These data confirmed that nicotine administration promotes plaque exosome retention.

To provide a solid theoretical foundation for the internalization of exosomes by VSMCs, we performed colocalization experiments of exosomes and VSMCs. α-SMA staining was performed in combination with CD9 staining, and the results were examined by confocal microscopy. VSMCs and exosomes were spatially clustered (Figure 1G), suggesting that VSMCs might be recipient cells of plaque exosomes.

Taken together, our results further confirmed that nicotine has pro-atherosclerotic effects and promotes intraplaque VSMC accumulation and exosome retention. These data suggest that in addition to the direct proatherosclerotic effects of nicotine on VSMCs, nicotine might also contribute to atherosclerotic lesion development by modulating the effect of exosomes on VSMCs.

### VSMCs internalize nicotine-induced macrophage-derived exosomes

To directly demonstrate that VSMCs internalize macrophage-derived exosomes, the first step was to isolate and purify exosomes. RAW264.7 macrophages were cultured with exosome-depleted medium with or without nicotine (10^-5^ M) for 48 h, and then, the supernatant was collected for subsequent analyses. M-Exos and NM-Exos were isolated by ultracentrifugation. Subsequently, sucrose density gradient fractionation was applied for exosome purification, and the vesicles were isolated in a subfraction at a density of 1.12 g/ml (Figure S1B). Interestingly, higher intracellular CD9 expression (Figure S1C) was detected in nicotine-stimulated macrophages. Nicotine-stimulated macrophages and control macrophages contributed 158.9 µg and 76.2 µg of exosomal protein per million cells, respectively, corresponding to a 2.09-fold difference (Figure S1D). This difference was further verified by analyzing total exosomal protein from the same number of control and nicotine-treated cells (Figure S1E). In addition, NTA of M-Exos and NM-Exos showed similar results to those based on total exosomal protein per million cells (Figure S1F and S1G). In brief, these data demonstrated that nicotine induces the biogenesis and secretion of macrophage-derived exosomes.

**Figure 2:**
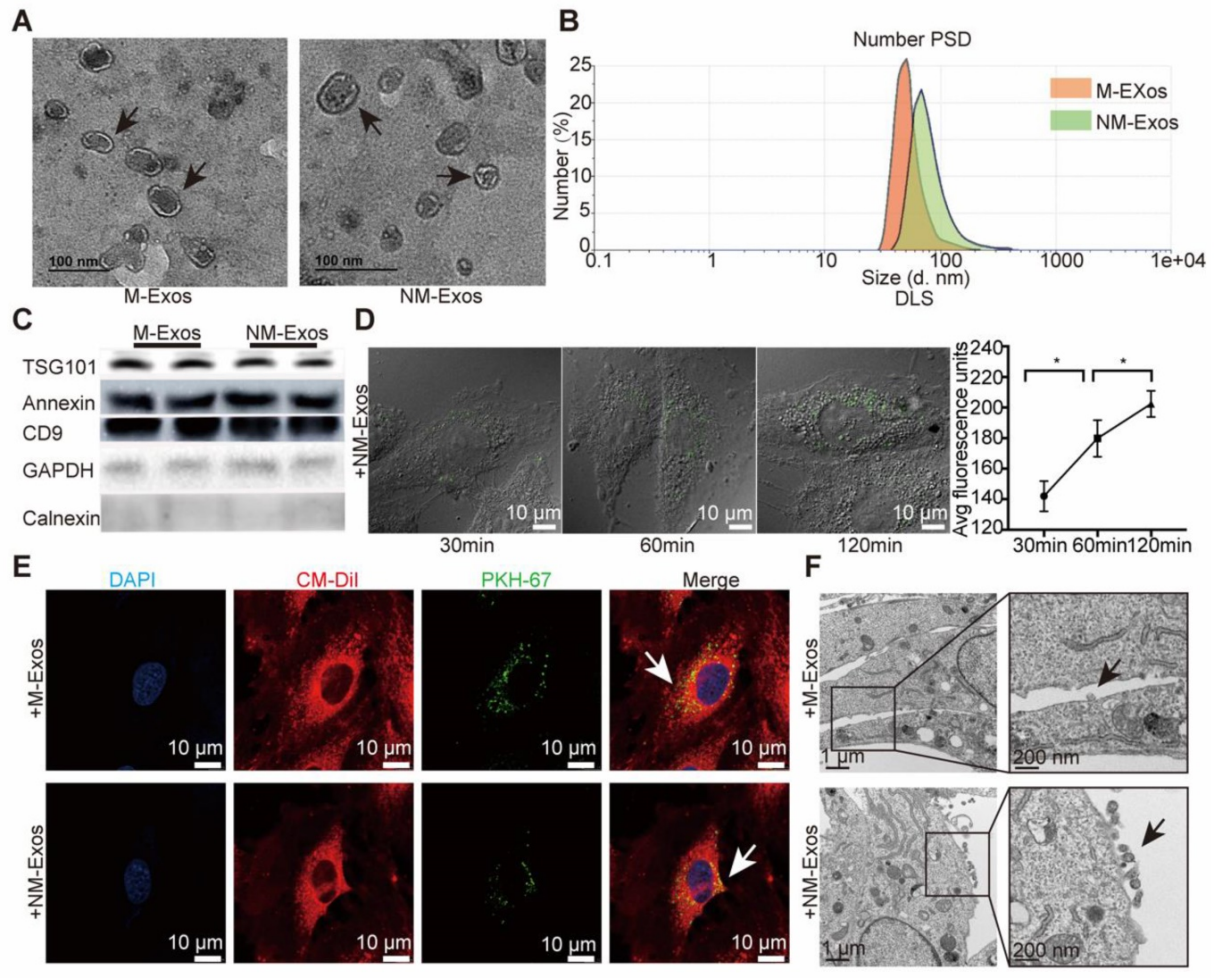
** Macrophage-derived exosome characterization.** (A) TEM of control macrophage-derived exosomes (M-Exos) and nicotine-induced macrophage-derived exosomes (NM-Exos). Magnification ×20000. (B) NTA of M-Exos and NM-Exos. (C) Western blotting of exosomal marker TSG101, Annexin II, CD9 and cell marker GAPDH and Calnexin. (D) Fluorescence images of VSMCs incubated with PKH67-labelled NM-Exos (green) for 30min, 60min and 120min and quantification of internalized exosomes were measured by average fluorescence units per sight (n=5). Magnification ×630. (E) Fluorescence images of VSMCs incubated with PKH67-labelled M-Exos or NM-Exos (green). DiI (red) was stained of membrane. Nuclei were stained with DAPI (blue). Magnification ×630. (F) TEM of VSMCs incubated with M-Exos and NM-Exos. Magnification ×11000 and ×20000. *P<0.05. All the data are presented as mean±SD (One-way *NOVA*).

TEM, DLS and western blotting analyses were performed to identify the purified nanoparticles derived from macrophages. TEM revealed that M-Exos and NM-Exos exhibited cup- or sphere-shaped morphology with double-membrane structures (Figure 2A), similar to previously described exosomes. DLS measurements showed that the diameter of these particles predominantly ranged from 30 nm to 100 nm, with peaks at 51 nm and 68 nm (Figure 2B), which was consistent with the previously reported exosome size distribution. The identity of these particles was further confirmed as exosomes by western blotting, which showed the presence of exosomal surface markers, including CD9, TSG101 and Annexin II (Figure 2C). In contrast, the cytoplasmic proteins calnexin and GAPDH showed low or absent expression in exosome lysates (Figure 2C). In addition, to exclude the interference of apoptotic debris, the apoptosis rate of macrophages used for exosome collection was confirmed to show no significant changes (Figure S2A and S2B) 31. All these data manifested that these nanoparticles were exosomes.

To demonstrate the uptake of exosomes, VSMCs were cocultured with PKH67-labelled exosomes and then visualized with confocal microscopy. The amount of internalized vesicles increased over time (Figure 2D and S2C), and the localization results showed that the vesicles surrounded the nuclei or lined the inner surface of the cell membrane after entering the cells, which implies that endocytosis might be the main mechanism through which VSMCs internalize exosomes (Figure 2E). In addition, TEM was used to directly observe the initiation of the uptake process (Figure 2F). The entire internalization process was video recorded for validation (Videos 1 and 2 in the supplementary material). In brief, these results provided solid evidence that VSMCs internalize macrophage-derived exosomes.

### Spatial and temporal distribution of nicotine-induced macrophage-derived exosomes

**Figure 3:**
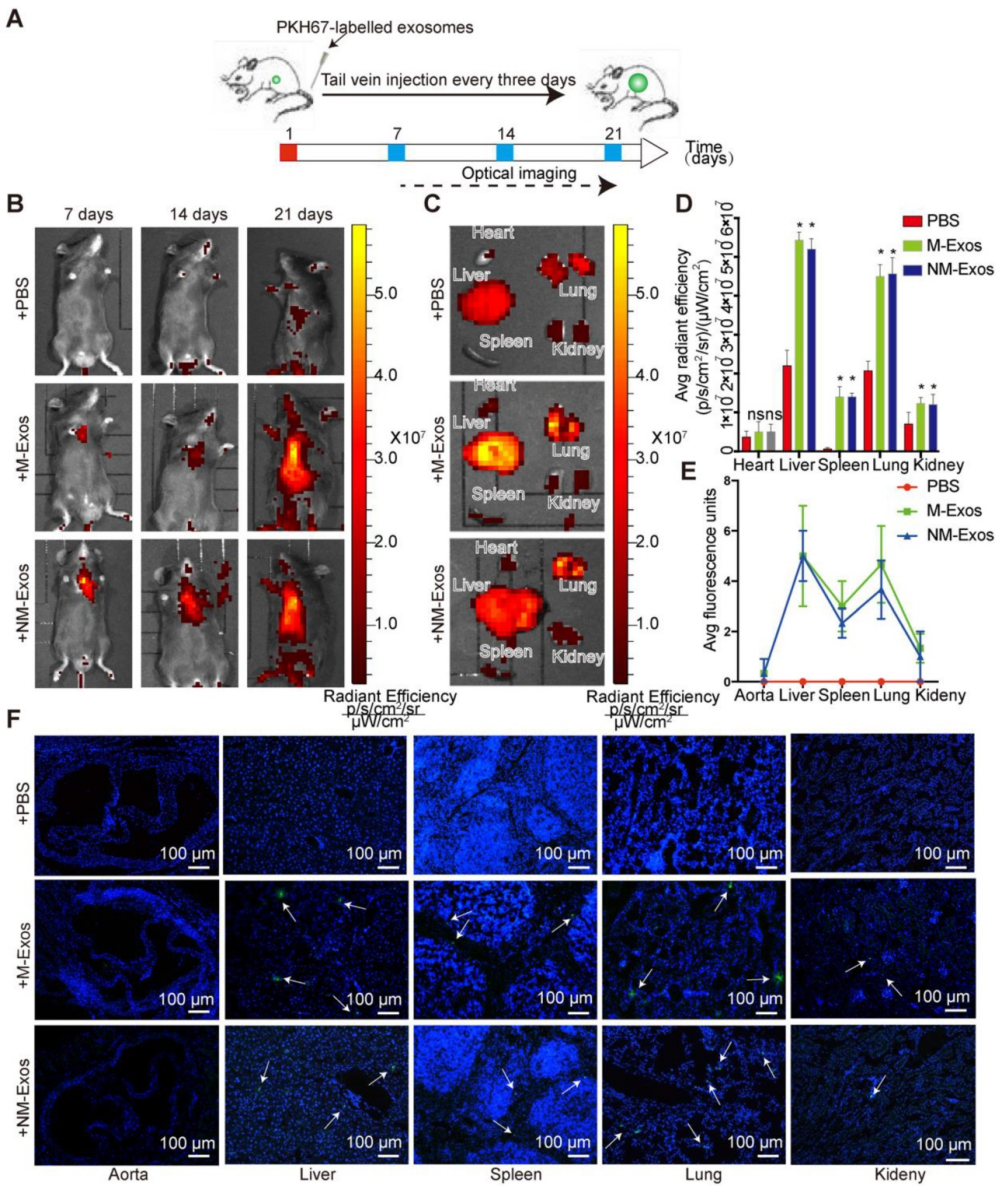
** Biodistribution of circulation macrophage-derived exosomes.** (A) Systematic administration of PKH67-labled exosomes in *ApoE*^-/-^ mice. (B) *In vivo* bioluminescence images to study the biodistribution of PKH67-labled M-Exos and NM-Exos in mice (n=6) at the indicated time points. (C) Representative *ex vivo* bioluminescence images of different organs. (D) Quantification of *ex vivo* bioluminescence in different organs (n=5). (E) Quantification of organ tissue average fluorescence units(n=5). (F) Images of PKH67-labled M-Exos and NM-Exos in organ tissue. Exosomes were labelled with PKH67 (green) and nuclei were stained with DAPI (blue). Magnification ×100. *P<0.05 vs PBS; ns, not significant. All the data are presented as mean±SD (One-way *NOVA*).

To elucidate the role of NM-Exos in intercellular communication and their targets, it was important to first determine the *in vivo* fate of exosomes. Here, we studied the chronic biodistribution of macrophage-derived exosomes in circulation in *ApoE*^-/-^ mice. Both NM-Exos and M-Exos were labelled with the lipid-associating fluorescence dye PKH67 and administered intravenously every three days for a total of 21 days (Figure 3A). To determine whether the tail vein injection was successful, *in vivo* epifluorescence was examined by the IVIS® system on days 7, 14 and 21. The results showed that epifluorescence increased with an increasing number of injections in both the NM-Exos and M-Exos groups (Figure 3B).

To demonstrate the general organ distribution of these two types of exosomes, *ex vivo* organ epifluorescence was examined on day 21. *Ex vivo* fluorescence quantification revealed significant accumulation of macrophage-derived exosomes in the lungs (1.9-fold increase), liver (2.3-fold increase) and spleen (8.8-fold increase) (Figure 3C and 3D). These results were further validated by microscopy. The tissue examination results showed greater exosomal fluorescence in the lungs and liver, not in the vessel wall (Figure 3E and 3F). Overall, the circulatory distribution results indicated that both sets of macrophage-derived exosomes showed highly similar biodistribution and retention in specific tissues and there would be little chance that the intraplaque retention exosomes were derived from circulation.

**Figure 4:**
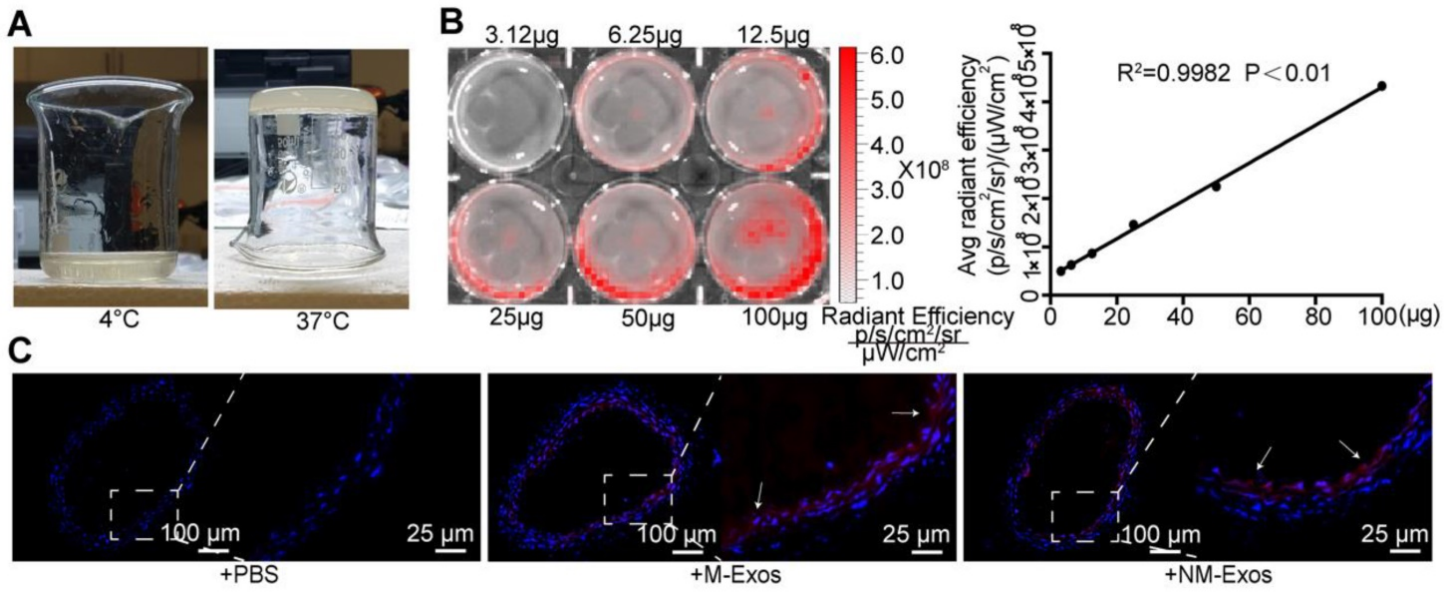
** Simulation of local secretion of macrophage-derived exosomes.** (A) Optical images of the chitosan solution (4℃) and hydrogel (37℃). (B) *Ex vivo* imaging of DiI-labelled exosomes showed increasing bioluminescence signals with concentrations of exosomes (R^2^=0.9982, P<0.01). (C) Fluorescent images of mouse abdominal aorta applied with thermosensitive chitosan hydrogel which incorporates DiI-labelled exosomes. Magnification ×100 and ×400.

Additionally, a model of local exosome secretion was designed to complement these results. To simulate local exosome secretion by macrophages, we placed thermosensitive gel coated, fluorescence-labelled exosomes around the abdominal aorta of *ApoE^-/-^* mice (Figure 4A and 4B) 22. The exosomes were gradually released and absorbed by neighboring tissue. More specifically, the exosomes readily distributed into the atrial wall and exhibited a strong signal in the whole atrial layer (Figure 4C). These data indicated that local exosome secretion results in exosome retention *in situ.*

The systemic administration and local modeling results indicated that exosomes within plaques are more likely to have been secreted locally.

### Nicotine-induced macrophage-derived exosomes promote VSMC migration and proliferation

**Figure 5:**
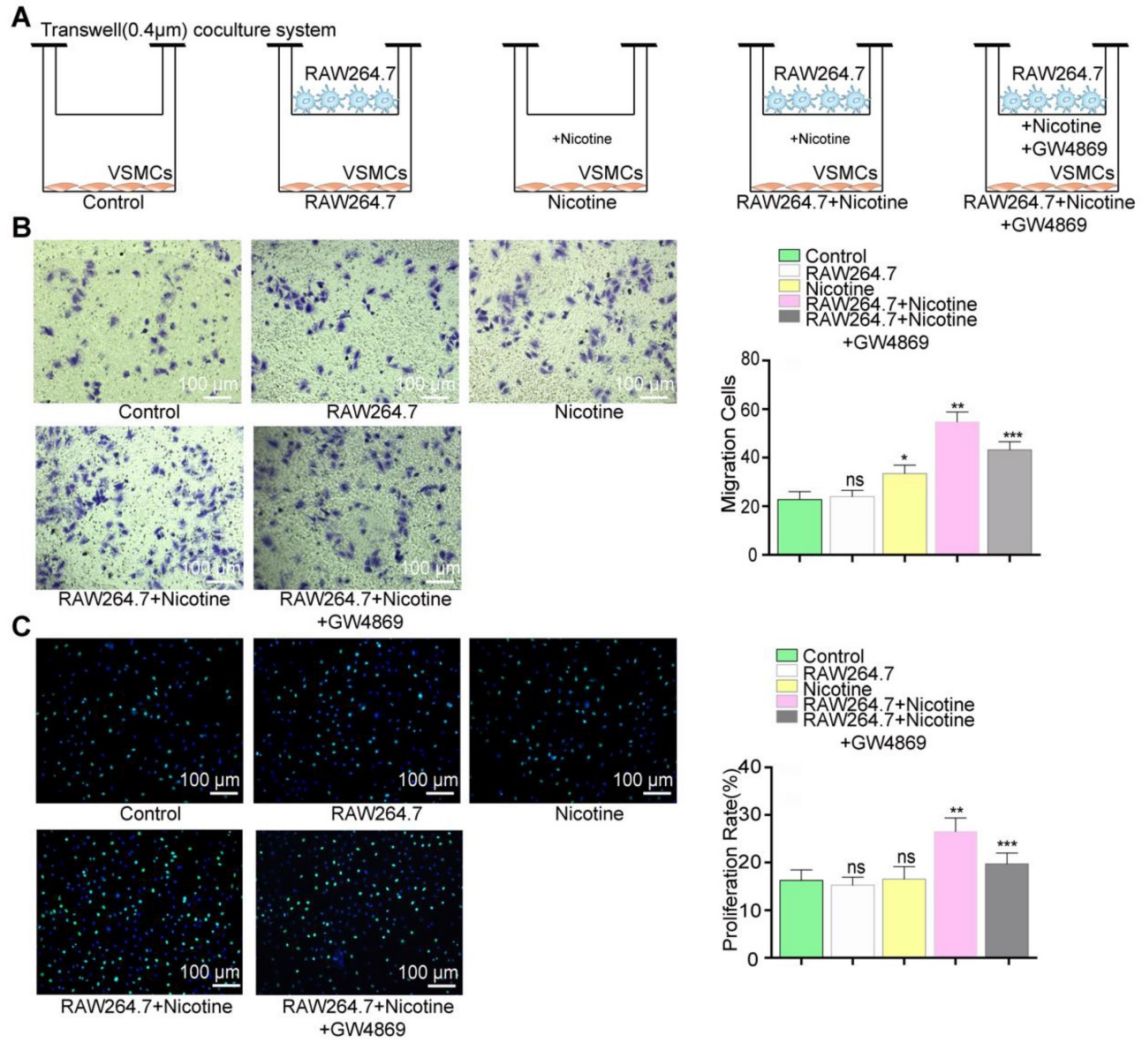
** Function evoke of VSMCs cocultured with nicotine-stimulated macrophages.** (A) VSMCs cocultured with macrophages in the presence or absence of nicotine. (B) Coculture with macrophages and nicotine (RAW264.7+Nicotine) promoted VSMCs proliferation and exosome inhibitor (RAW264.7+Nicotine +GW4869) erased this effect in the transwell (8um) assay (n=5). Magnification ×100. (B) Coculture with macrophage and nicotine (RAW264.7+Nicotine) promoted VSMCs migration and exosome inhibitor (RAW264.7+Nicotine +GW4869) earased this effect in the EdU staining (n=5). Magnification ×100. *P<0.05 vs Control, **P<0.05 vs Nicotine, ***P<0.05 vs RAW264.7+Nicotine; ns, not significant. All the data are presented as mean±SD (One-way *NOVA*).

To better understand the crosstalk between nicotine-stimulated macrophages and VSMCs, a transwell coculture system (0.4 µm) was used to simulate this intercellular communication (Figure 5A). In brief, we cocultured macrophages and VSMCs in the presence or absence of nicotine (10^-5^ M) for 24 h and then examined functional alterations in the VSMCs. The atherogenic phenotype of VSMCs is invariably associated with increased cell migration and proliferation, so we examined these two cellular processes.

VSMCs incubated with nicotine-stimulated macrophages showed markedly increased migration (36% in the transwell assay) and proliferation (39% in the EdU staining) compared to those cocultured with only macrophages or nicotine (Figure 5B and 5C). Therefore, the data indicated that nicotine-treated macrophages can activate VSMC proliferation and migration via cell-to-cell communication.

However, the participation of NM-Exos remained uncertain. Thus, GW4869, a confirmed exosome secretion inhibitor, was added into the culture media 19, 32. Figures S2D and S2E showed exosome retention in RAW264.7 macrophages with or without nicotine stimulation, suggesting successful inhibition of exosome secretion by GW4869. The effects of nicotine-treated macrophages were alleviated by GW4869, as evidenced by the 37% decrease in migration in the transwell assay (Figure 5B) and the 40% decrease in proliferation in the EdU staining (Figure 5C). These results indicate that macrophage-derived exosomes regulate VSMC proliferation and migration.

**Figure 6:**
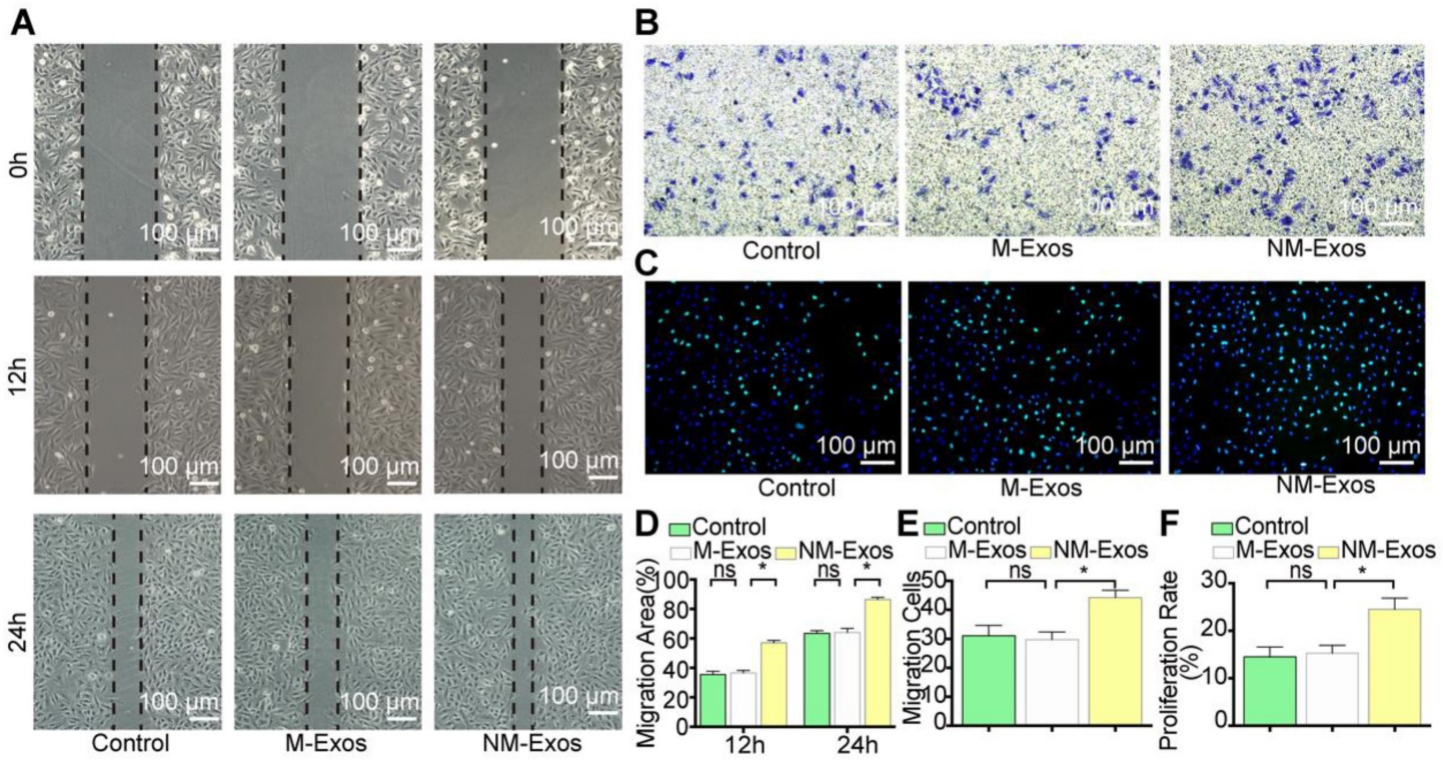
** NM-Exos promote VSMCs proliferation and migration.** (A) NM-Exos promoted VSMCs migration measured by scratch wound assay. Magnification ×100. (B) NM-Exos promoted VSMCs migration measured by transwell (8um) assay. Magnification ×100. (C) NM-Exos promoted VSMCs proliferation measured by EdU staining. Magnification ×100. (D) Quantitative analysis of the migration area in (A) (n = 5). (E) Quantitative analysis of the migration cells in (B) (n = 5). (F) Quantitative analysis of the proliferation rate in (C) (n = 5). *P<0.05; ns, not significant. All the data are presented as mean±SD (One-way *NOVA*).

To confirm the modulatory role of NM-Exos, VSMCs were cocultured with purified NM-Exos. The results confirmed that NM-Exos significantly increased VSMC migration (31% at 12 h and 27% at 24 h in the scratch wound assay in Figure 6A and 6D, and 31% in the transwell assay in Figure 6B and 6E) and proliferation (35% in the EdU staining in Figure 6C and 6F) compared to M-Exos, which was in agreement with the coculture results. Overall, this series of studies led to the conclusion that NM-Exos encourage VSMC migration and proliferation.

### Differential miRNA expression profile in nicotine-induced macrophage-derived exosomes

**Figure 7:**
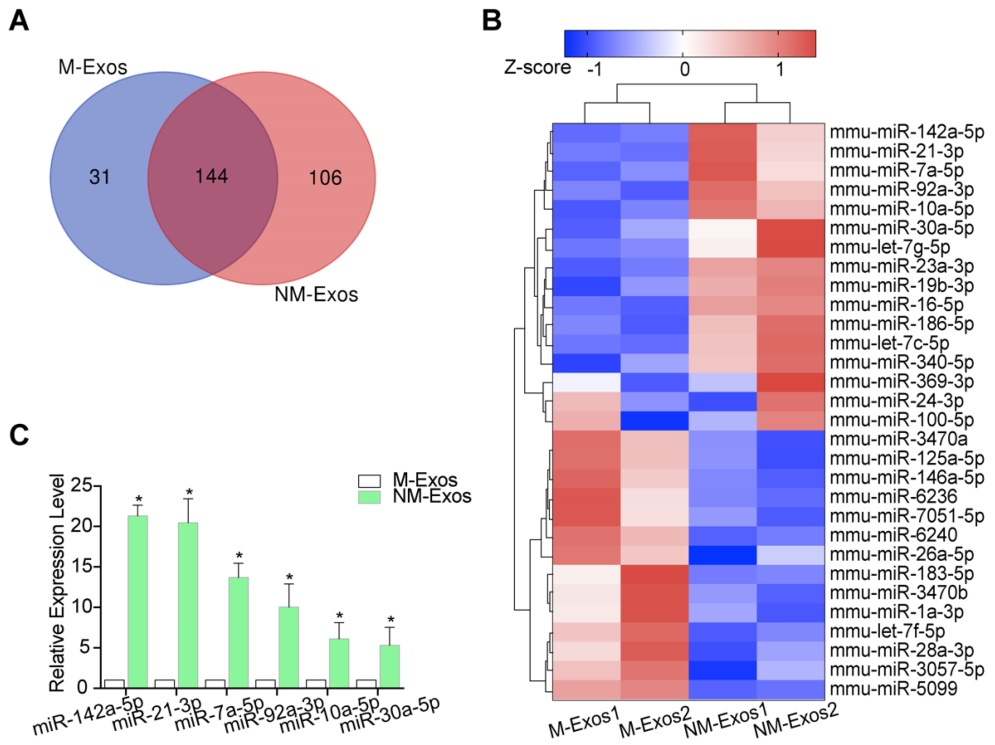
** miRNA expression profiles of NM-Exos. (**A) Venn diagram showing the unique and overlapping miRNAs presented in M-Exos and NM-Exos. (B) Heatmap showing Z-scores of miRNAs from M-Exos and NM-Exos (fold change≥2 or ≤0.05, FDR<0.05). Red represents up-regulated genes and blue represents down-regulated genes. (C) qRT-PCR validated the increased amount of miR-142a-5p, miR-21-3p, and miR-7a-5p, etc in NM-Exos compared to in M-Exos (n=3). *P<0.05. All the data are presented as mean±SD (*Student's t-test*).

Emerging evidence supports the theory that exosomal miRNAs function as important regulators of cell function 30, 33. To determine the mechanism by which NM-Exos mediate VSMC migration and proliferation, we profiled the miRNAs in NM-Exos in two replicates using high-throughput sequencing (miRNA-seq); M-Exos were also profiled as a comparative sample. A total of 250 and 175 known mature miRNAs were identified in NM-Exos and M-Exos, respectively (Figure 7A). Among these miRNAs, 144 were detected in both NM-Exos and M-Exos (Figure 7A).

To compare miRNA expression levels between M-Exos and NM-Exos, cluster analysis based on 30 differentially expressed miRNAs (FDR<0.05, NM-Exos/M-Exos ≥2 or ≤0.05) was used to generate a tree with a clear distinction between the two groups (Figure 7B). Compared to M-Exos, NM-Exos showed the enrichment of 10 mature miRNAs (Figure 7B). Two miRNAs (miR-142a-5p and miR-21-3p) that were enriched in NM-Exos were reported to participate in the regulation of cell migration and proliferation 34, 35.

To validate these miRNA-seq results, the expression of six selected miRNAs in NM-Exos was measured by qRT-PCR. Consistent with the miRNA-seq results, qRT-PCR showed that miR-142a-5p, miR-21-3p, miR-7a-5p, miR-92a-3p, miR-10a-5p and miR-30a-5p were enriched in NM-Exos compared to M-Exos (Figure 7C).

### miR-21-3p is secreted in exosomes

Our results showed that macrophages secreted exosomes enriched with miRNAs, including miR-142a-5p and miR-21-3p. Unlike miR-142a-5p, miR-21-3p was reported to participate in vascular injury and repair 36, 37. Thus, we focused on miR-21-3p in the subsequent experiments.

**Figure 8:**
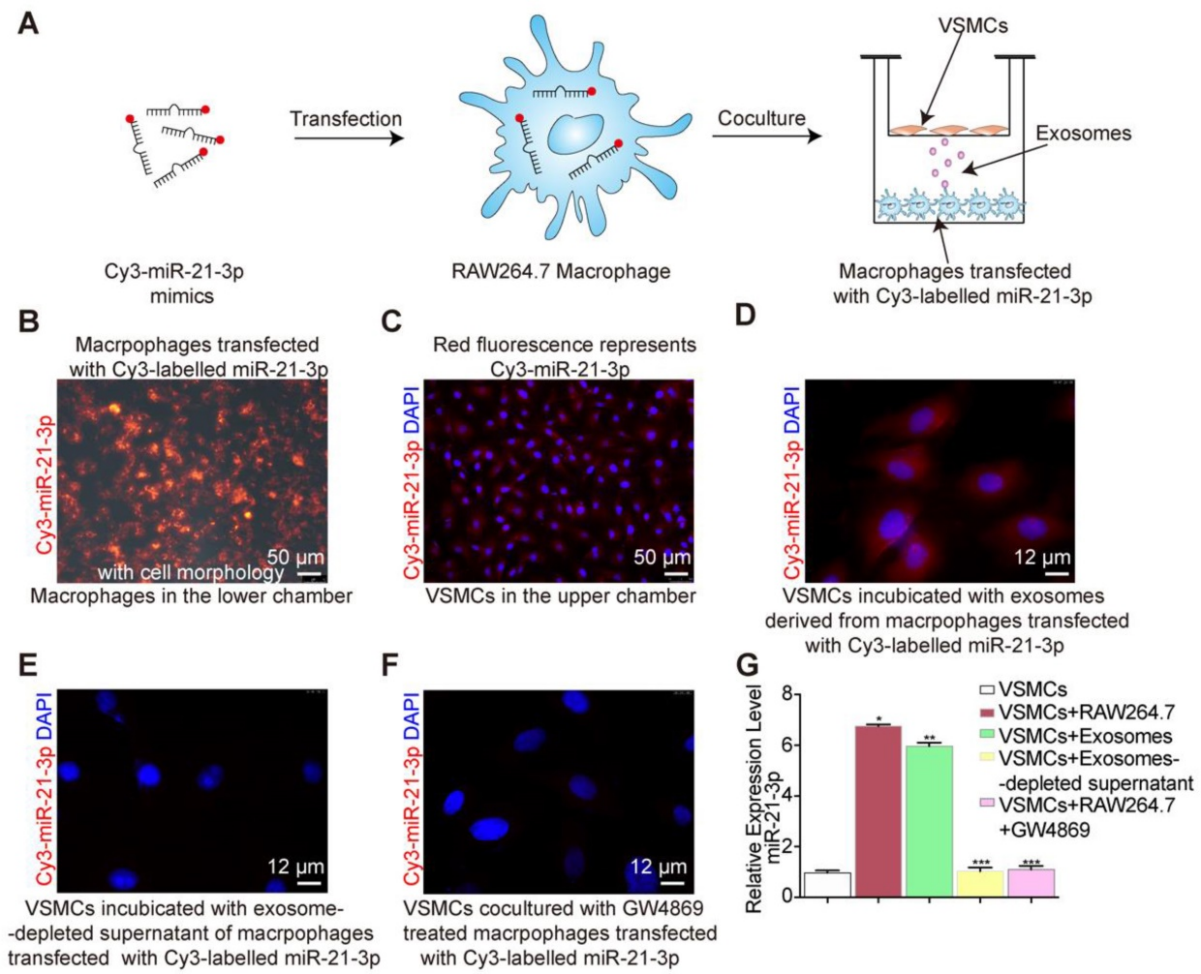
** Exosomes shuttle miR-21-3p. (**A) RAW264.7 macrophages transfected with a Cy3-labelled miR-21-3p mimics (red) were cocultured with VSMCs in a transwell (0.4μm) plate. (B) Representative image of RAW264.7 macrophages transfected with a Cy3-labelled miR-21-3p mimics with cell morphology. Magnification ×100. (C) Fluorescence image of VSMCs in the upper transwell (0.4μm) chamber. Magnification ×100. (D) Fluorescence image of VSMCs incubated with exosomes derived from macrophages transfected with Cy3-miR-21-3p. Magnification ×630. (E) VSMCs incubated with exosome-depleted supernatant of macrophages transfected with Cy3-miR-21-3p. Magnification ×630. (F) RAW264.7 macrophages transfected with a Cy3-labelled miR-21-3p mimics pre-treated by GW4869 (10μM) were cocultured with VSMCs in a transwell (0.4μm) plate. Magnification ×630. (G) Relative expression level of miR-21-3p of each group confirmed by qRT-PCR. Experiments were performed in triplicate (n=3). *P<0.05, **P<0.05 vs VSMCs, ***P<0.05 vs VSMCs+RAW264.7/ VSMCs+Exosomes. All the data are presented as mean±SD (One-way *NOVA*).

After nicotine stimulation, miR-21-3p is packaged into exosomes and then transferred to recipient cells, yet it was not clear whether miR-21-3p is mainly transferred by exosomes. To identify the miR-21-3p delivery methods, Cy3-labelled miR-21-3p mimics (red fluorescence) were used to trace the intercellular transport of miR-21-3p and further study this form of communication (Figure 8A). We transfected macrophages in the lower chamber with Cy3-labelled miR-21-3p mimics (100 nM) (Figure 8B) and then cocultured them with VSMCs in the upper chamber for 24 h in a transwell apparatus (0.4 μm) (Figure 8A). The appearance of red fluorescence in VSMCs indicated the intercellular transfer of miR-21-3p derived from the transfected macrophages (Figure 8C). After incubation with isolated exosomes from transfected macrophages, VSMCs showed red fluorescence (Figure 8D), whereas little red fluorescence was observed after transfection with exosome-depleted supernatant (Figure 8E). These data suggested that miR-21-3p can be delivered by exosomes. In addition, when macrophage-derived exosome secretion was blocked by GW4869, the red fluorescence signal significantly decreased (Figure 8F). Consistent with these tracing results, miR-21-3p expression levels were in agreement with the fluorescence changes in VSMCs (Figure 8G). Taken together, these results suggested that miR-21-3p is mostly secreted in the form of exosomes.

### Nicotine-induced macrophage-derived exosomal miR-21-3p promotes VSMC migration and proliferation

**Figure 9:**
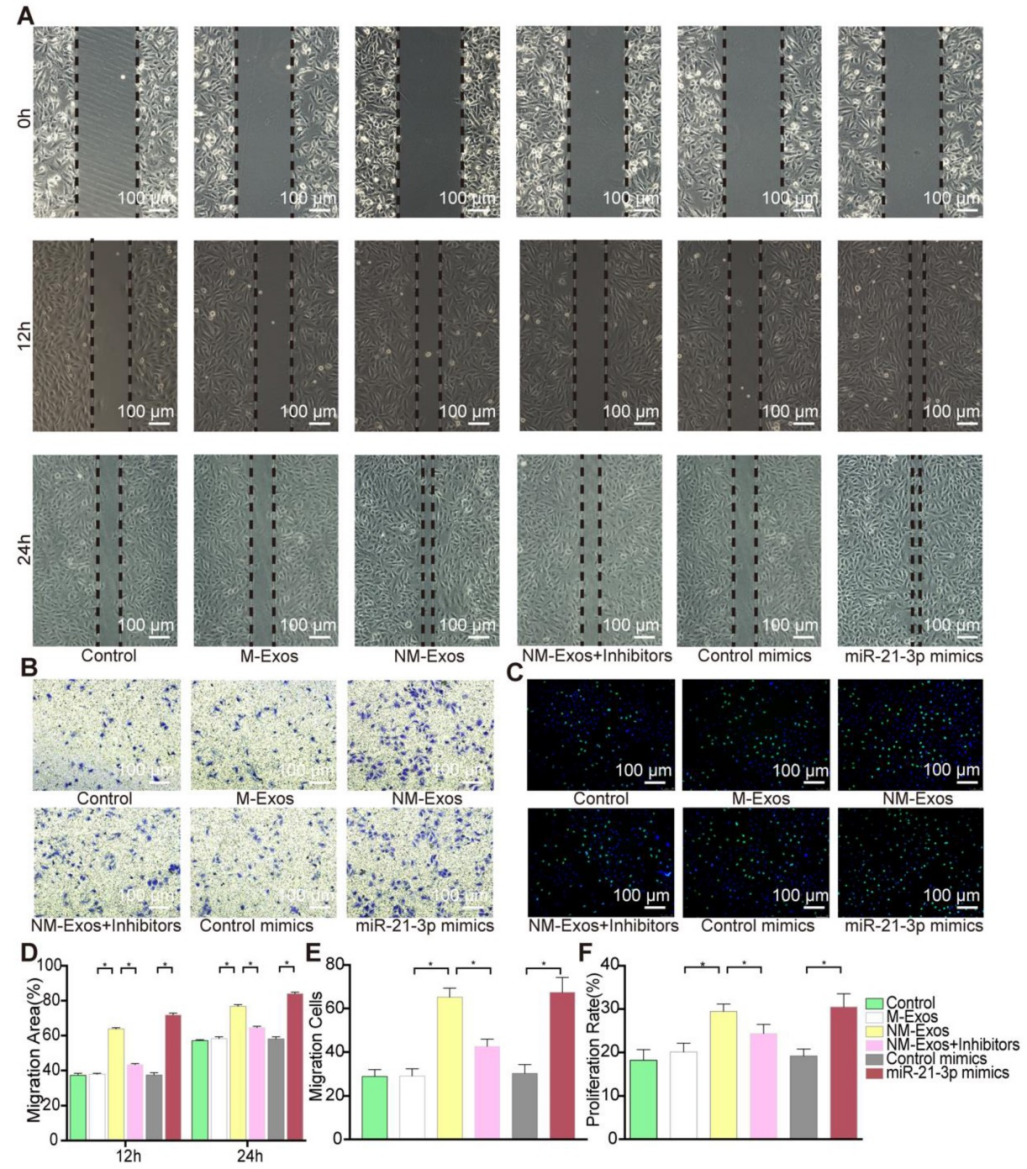
** miR-21-3p is the major function molecular in NM-Exos. (**A) NM-Exos and miR-21-3p mimics promoted VSMCs migration but this effect was impaired by miR-21-3p inhibitors (NM-Exos+Inhibitors) in wound scartch assay. Magnification ×100. (B) miR-21-3p inhibitors (NM-Exos+Inhibitors) attenuated NM-Exos effect on VSMCs in transwell(8μM) assay. Magnification ×100. (C) miR-21-3p inhibitors (NM-Exos+Inhibitors) attenuated NM-Exos effect on VSMCs in EdU staining. Magnification ×100. (D) Quantitative analysis of the migration rates in (A) (n = 5). (E) Quantitative analysis of the migration rates in (B) (n = 5). (F) Quantitative analysis of the proliferation rates in (C) (n =5). *P<0.05. All the data are presented as mean±SD (One-way *NOVA*).

We postulated that miR-21-3p might partially mediate the effects of NM-Exos on VSMCs. miR-21-3p mimics and miR-21-3p inhibitors were utilized in VSMC functional assays. After miR-21-3p mimic transfection, VSMCs showed increased migratory ability in the scratch wound assay (80% at 12 h and 25% at 24 h, as shown in Figure 9A and 9D), and these results were further validated by the transwell assay (26% increase, as shown in Figure 9B and 9E). Additionally, miR-21-3p mimic-transfected VSMCs exhibited a 22% increase in proliferation in the EdU staining (Figure 9C and 9F). These data indicated that miR-21-3p overexpression and NM-Exos promote similar changes in VSMCs.

Moreover, the absence of miR-21-3p impaired the pro-migratory and pro-proliferative effects of NM-Exos on VSMCs. Compared to VSMCs incubated with NM-Exos only, VSMCs transfected with miR-21-3p inhibitors and incubated with NM-Exos presented decreases in migration of 52% (at 12 h) and 35% (24 h) in the scratch wound assay (Figure 9A and 9D) and of 47% in the transwell assay (Figure 9B and 9E). In addition, miR-21-3p inhibition caused a significant 20% growth suppression in the EdU staining (Figure 9C and 9F). These results further confirmed the role of miR-21-3p in NM-Exos-treated VSMCs.

Overall, the effects of NM-Exos and miR-21-3p on VSMCs were similar, and the outcomes in VSMCs evoked by NM-Exos were attenuated by miR-21-3p knockdown, indicating that NM-Exos promote VSMC migration and proliferation through miR-21-3p.

### Inhibition of PTEN induces miR-21-3p-like effects in VSMCs

To elucidate the molecular mechanism by which miR-21-3p participates in VSMC migration and proliferation, TargetScan, miRWalk and miRbase were used to predict target mRNAs 38. The mRNAs predicted by all these algorithms were selected for further analysis. Among the potential targets of miR-21-3p, PTEN has been studied in the context of vascular remodeling, cell migration and proliferation 39-42.

**Figure 10:**
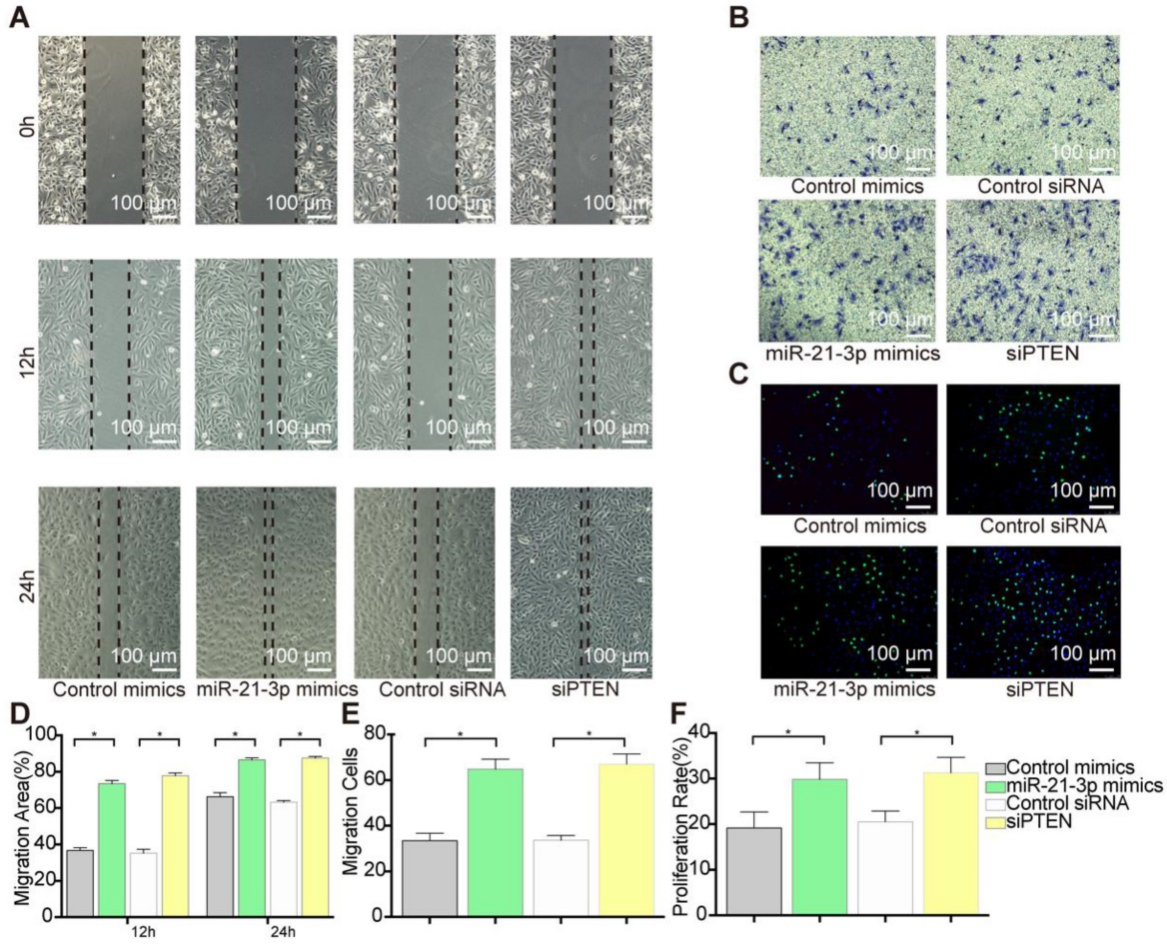
** VSMCs function alternation after PTEN inhibition.** (A) siPTEN promoted VSMCs migration as miR-21-3p in scratch wound assay. Magnification ×100. (B) siPTEN promoted VSMCs migration as miR-21-3p in transwell (8μm) assay. Magnification ×100. (C) siPTEN promoted VSMCs proliferation as miR-21-3p in EdU staining. Magnification ×100. (D) Quantitative analysis of the migration area of scratch wound assay (n=5) in (A). (E) Quantitative analysis of the migration cells of transwell (8μm) assay (n=5) in (B). (F) Quantitative analysis of the proliferation rate in EdU staining (n=5) in (C). *P<0.05. All the data are presented as mean±SD (*Student's t-test*).

To examine the role of PTEN in VSMC migration and proliferation, we selectively knocked down PTEN using specific siRNA. Transfection with siPTEN resulted in 78% (at 12 h) and 27% increases (at 24 h) in the wound healing area (Figure 10A and 10D) and a 31% increase in cell migration, which indicated a significant promotion of migration (Figure 10B and 10E). Similarly, PTEN suppression increased VSMC proliferation by 25% in the EdU staining (Figure 10C and 10F). These data suggested that PTEN inhibition contributes to VSMC migration and proliferation.

According to the predictions, PTEN is targeted by miR-21-3p for downregulation, suggesting that PTEN and miR-21-3p should show opposite expression profiles during VSMC migration and proliferation. Thus, to verify the correlation between miR-21-3p and PTEN, miR-21-3p mimics were transfected into VSMCs to overexpress miR-21-3p. Similar to the effects of PTEN interference, miR-21-3p overexpression resulted in increases in migration of 74% (at 12 h) and 22% (at 24 h) in the scratch wound assay (Figure 10A and 10D) and of 27% in the transwell assay (Figure 10B and 10E) and increases in proliferation of 21% in the EdU staining (Figure 10C and 10F). Overall, miR-21-3p might regulate VSMC migration and proliferation via the inhibition of PTEN.

### miR-21-3p mediates the proatherogenic effect of nicotine-induced macrophage-derived exosomes on VSMC function by targeting PTEN

**Figure 11:**
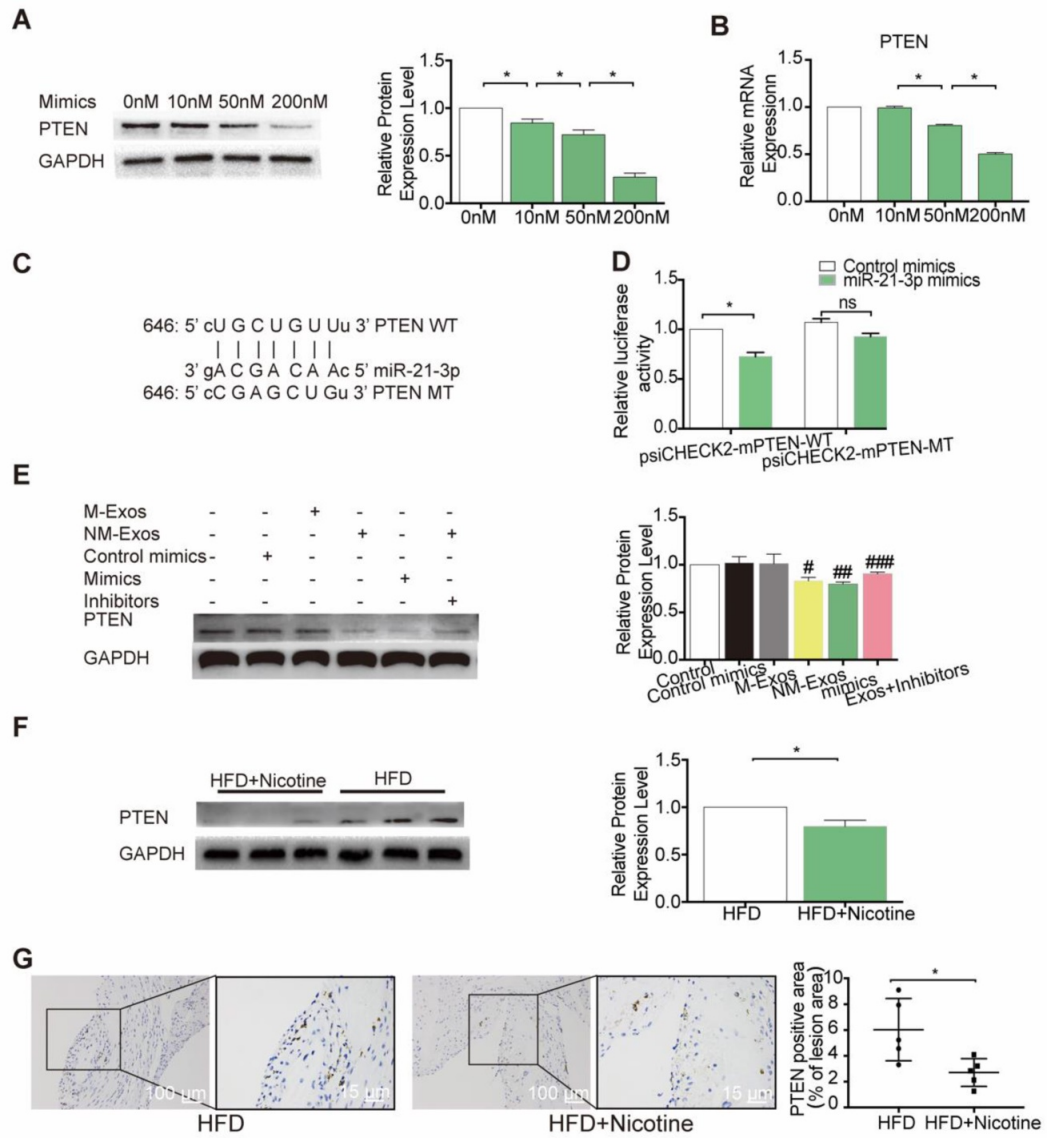
** miR-21-3p regulates PTEN expression *in vitro* and *in vivo*. (**A) Expression level of PTEN protein in VSMCs after transfected with different concentration of miR-21-3p mimics (n=3). (B) miR-21-3p blocked the expression of PTEN in VSMCs at the transcriptional level in a dose dependent manner (n=3). (C) Predicted interaction between miR-21-3p and its putative binding sites in the 3′UTR of PTEN. (D) Normalized luciferase activity 48h after co-transfection of control mimics or miR-21-3p mimics together with psiCHECK2-mPTEN-WT or psiCHECK2-mPTEN-MUT (n=3). (E) Total PTEN protein expression in different treatment (n=3). (F) Expression level of PTEN protein in HFD and HFD+Nicotine group mouse aorta (n=3). (G) Immunohistochemistry was stained for PTEN in HFD and HFD+Nicotine group aortic root atherosclerotic lesion (n=5). Magnification ×100 and ×400. *P <0.05, #P<0.05 vs M-Exos, ##P<0.05 vs Con mimics, ###P<0.05 vs NM-Exos; ns, not significant. All the data are presented as mean±SD (One-way *NOVA* and* Student's t-test*).

To further identify the modulatory role of miR-21-3p on PTEN, VSMCs were treated with a concentration gradient of miR-21-3p mimics (0 nM, 10 nM, 50 nM and 200 nM), and PTEN expression was compared at both the mRNA and protein levels. The results demonstrated that both PTEN mRNA and protein were repressed by miR-21-3p in a dose-dependent manner (Figure 11A and 11B). Finally, to validate whether PTEN is a target gene of miR-21-3p, we performed dual-luciferase reporter assay and identified the miR-21-3p binding site in PTEN, which was predicted by TargetScan to be in the 3'UTR (Figure 11C). Luciferase reporters that contained either wild-type (psiCHECK2-PTEN-WT) or mutated (psiCHECK2-PTEN-MUT) PTEN 3′UTR were transfected into HEK-293 cells, along with either miR-21-3p mimics or control mimics (Figure 11D). When cells were transfected with miR-21-3p mimics, luciferase reporter activity decreased. In contrast, when the PTEN 3′UTR mutant was transfected into cells, there was no effect on luciferase activity. These data indicated that miR-21-3p can directly target the 3′UTR of PTEN to regulate VSMC function.

To identify whether NM-Exos influence the expression of PTEN, VSMCs were cocultured with NM-Exos. The results showed that NM-Exos reduced PTEN expression, indicating that NM-Exos can also regulate the expression of this protein (Figure 11E). To validate a pivotal role of miR-21-3p in the functions of NM-Exos, NM-Exos-treated VSMCs were transfected with miR-21-3p inhibitors. VSMCs treated with miR-21-3p inhibitors and M-Exos were used as a negative control, and VSMCs treated with miR-21-3p mimics were used as a positive control. VSMCs treated with miR-21-3p inhibitors failed to suppress PTEN expression. Furthermore, western blotting of aortic tissue in the HFD and HFD+nicotine groups showed reduced PTEN expression, and these findings were validated by immunohistochemistry (Figure 11F and 11G). In summary, miR-21-3p mediates the proatherogenic effect of NM-Exos on VSMC function by targeting PTEN.

## Discussion

This study demonstrates that nicotine exposure modulates cell-to-cell communication between macrophages and VSMCs, which promotes VSMC migration and proliferation via the targeting of PTEN by exosomal miR-21-3p, thereby accelerating plaque progression. Therefore, targeting miR-21-3p in combination with existing conventional therapies might be a novel therapeutic strategy for nicotine-associated atherosclerosis.

Nicotine is one of the major ingredients of cigarette smoke and is considered the major contributor to cardiovascular disease risk. Although previous studies have proven that nicotine can enhance the migration and proliferation ability of VSMCs in various stages of atherogenesis 13, 15, based on our research and analysis, these studies are unsatisfactory and inadequate. In this regard, our study better complements the results that nicotine not only increases plaque VSMCs but also promotes this phenomenon through exosome secretion.

However, there are still some uncertainties about the potential sources of increased levels of exosomes. In atherosclerosis, there is no doubt that macrophages should be the first focus due to their pivotal role in disease initiation and progression. Macrophages are first recruited to the intima and turn into foam cells, and they extensively secrete various substances that influence neighboring cells in all stages of atherosclerosis 9. Previous studies showed that inflammation induces exosome secretion, yet whether nicotine, an inflammatory stimulator, has the same effect was not clear 7. Such results are more convincing and comprehensive considering the findings from our study: nicotine exposure enhanced exosome secretion by macrophages. Moreover, considering the similar extent of exosome retention in the nicotine group both *in vivo* and *in vitro*, it is reasonable to conclude that macrophages are likely the major source of increased exosomes in plaques.

Additional studies have recently reported that macrophage-derived extracellular vesicles isolated from atherosclerotic plaques are also involved in the progression of atherosclerosis 43, 44. In an effort to elucidate the role of NM-Exos in intercellular communication and their targets, first and foremost, it was necessary to determine the *in vivo* fate of exosomes. Thus, we established two models to explore the probable biodistribution of macrophage-derived exosomes, which, as expected, provided rational for the following study. First, we fluorescently labelled macrophage-derived exosomes and showed their circulatory fate using the IVIS® Spectrum system and tissue tracing techniques. The results indicated a slim chance that exosomes retained by the atrial wall are of circulatory origin. The large amounts of labelled exosomes (20 mg exosomes/mouse/3 days) that we injected were mostly retained in the liver, lungs and spleen, which are mainly recognized as blood filtration organs. However, other filtration organs, such as the kidneys, did not harbor many exosomes. A common characteristic of the liver, lungs and spleen is the abundance of tissue-resident phagocytes, such as Kupffer cells in the liver, alveolar macrophages in the lungs and macrophages in the spleen, while the kidney lacks such cells. This phenomenon also exists for breast cancer-derived exosomes 21. These data indicated that phagocytes might be a natural target of exosomes regardless of cell origin. The artery itself is not a filtration organ, meaning that blood flow is much faster in the aorta than in filtration organs. Therefore, we speculated that it would be difficult for exosomes to enter the artery wall.

Afterwards, due to the former conclusion that macrophages are more likely to be the major source of the increase in plaque exosomes, we designed a model of local exosome secretion to confirm the previous results. We applied thermosensitive gel-coated fluorescence-labelled exosomes to the area surrounding the abdominal aorta of *ApoE*^-/-^ mice, and these exosomes were gradually released to simulate the *in situ* exosome secretory process 22. Together with the systemic exosome administration results, these results indicated that exosomes retained in the artery probably originate from macrophages via *in situ* secretion. Additionally, according to the paracrine characteristics of exosomes, these macrophage-derived exosomes likely function at a short distance 8, 19. Overall, we mainly focused on macrophages as the source of exosomes and conducted studies based on the hypothesis that nicotine promotes VSMC migration and proliferation by inducing macrophage-derived exosomes.

Atherogenic macrophages release exosomes, which participate in mediating intercellular communication with VSMCs. According to previous studies, these exosomes can activate VSMC phenotype switching 7, 45. Nevertheless, it is unclear whether nicotine exposure augments atherosclerosis by inducing macrophage-derived exosomes in VSMCs. In our study, a series of related *in vitro* assays showed that NM-Exos stimulation of VSMCs produced similar results as coculture, with both conditions promoting migration and proliferation. Therefore, it can be concluded that NM-Exos can mediate cell-to-cell communication between macrophages and VSMCs.

Exosomes containing large amounts of miRNAs and serve as vehicles to transfer miRNAs to recipient cells; these exogenous miRNAs function as inhibitors of target gene expression by inducing translational repression and mRNA degradation 46. The miRNA-seq results showed that miR-21-3p was enriched in exosomes secreted by nicotine-stimulated macrophages. Further observations revealed that miR-21-3p was expressed in macrophages and then secreted to surrounding VSMCs in exosomes, which promoted atherosclerosis 8.

As one of the most well-studied miRNAs, miR-21-3p has been implicated in promoting invasion in various types of cancer 47-49 and has been reported to participate in vascular injury and repair 36, 37. The evidence points to a possibility that miR-21-3p may facilitate VSMC regeneration in atherosclerosis. Our results have confirmed the hypothesis that miR-21-3p is a modulator of the increased proliferation and migration of VSMCs. The possible mRNA targets of miR-21-3p were predicted by TargetScan, miRWalk and miRbase. Among all candidate mRNAs, PTEN has been studied in the context of VSMC migration and proliferation 40-42. Dual-luciferase assay was performed to confirm whether miR-21-3p can bind to the predicted binding site in the PTEN 3'UTR. The positive results substantiated our hypothesis. Additionally, western blotting and immunohistochemistry were utilized to detect PTEN expression. Taken together, the results arguably showed that nicotine promotes atherosclerosis progression by affecting VSMC proliferation and migration through the targeting of PTEN by macrophage-derived exosomal miR-21-3p.

In conclusion, this study revealed cell-cell communication between nicotine-treated macrophages and VSMCs that promotes atherogenesis. Thus, our study reveals a new mechanism of nicotine-induced atherosclerosis: nicotine increases VSMC migration and proliferation via a previously undescribed macrophage → exosomal miR-21-3p → PTEN pathway (Graphic abstract). More importantly and practically, this essential communication by exosomes, which connect macrophages and VSMCs, may provide novel options for preventive therapies in the future and may facilitate the development of personalized diagnostics and therapeutics for patients with smoking-associated atherosclerosis.

## Supplementary Material

Supplementary figures and tables.

Video 1.

Video 2.

## Abbreviations

VSMC: vascular smooth muscle cells
miRNA: microRNA
HFD: high-fat diet
PBS: phosphate buffer saline
HE: hematoxylin and eosin
α-SMA: α-smooth muscle actin
M-Exos: exosomes derived from control macrophages
NM-Exos: exosomes derived from nicotine-induced macrophages
qRT-PCR: quantitative real-time polymerase chain reaction
PTEN: phosphatase and tension homologue
NCD: normal chow diet
OCT: optimal cutting temperature compound
DLS: dynamic light scattering
PSD: particle size distribution
NTA: nanoparticle tracing analysis
TEM: Transmission electron microscopy
OsO4: osmium tetroxide
TBE: Tris-borate-EDTA
β-GP: β-glycerophosphate
CPM: counts per million
SDS-PAGE: sulfate-polyacrylamide gel electrophoresis
SD: standard deviation
miRNA-seq: high-throughput miRNA sequencing
