# Supplementary material for: Exosomes from nicotine-stimulated macrophages accelerate atherosclerosis through miR-21-3p/PTEN-mediated VSMC migration and proliferation
Source: Theranostics. 2019 Sep 21;9(23):6901–19. doi: 10.7150/thno.37357 (PMC6815950; doi:10.7150/thno.37357)
Supplement: Supplementary file 1 — Supplementary figures and tables. [file thnov09p6901s1.pdf]

Table S1.

	NCD	HFD	HFD + Nicotine
Weight gain, g	8.36±1.945	5.37±2.131*	1.86±1.235*#
Systolic pressure, mmHg	99.73±5.172	107.22±7.612*	117.30±4.03*
Diastolic pressure, mmHg	78.03±7.593	86.00±7.626*	94.60±5.211*
Triglycerides, mmol/L	0.946±0.305	0.791±0.197*	0.801±0.221*
Total cholesterol, mmol/L	20.376±2.918	36.927±3.174*	36.294±4.789*
HDL-C, mmol/L	1.929±0.182	2.833±0.317*	2.817±0.448*
LDL-C, mmol/L	10.263±0.690	9.729±0.647	10.101±1.636
Fast blood glucose, mmol/L	5.76±0.709	7.47±1.163*	7.33±0.952*
Blood uric acid, μmol/L	94.867±36.428	106.000±26.477*	121.100±55.347*

Table S1. The baseline characteristics of *ApoE$^{-/-}$* mice. NCD, Normal chow diet, HFD, High-fat diet. HDL-C, High-density lipoprotein cholesterol, LDL-C, Low-density lipoprotein cholesterol. *p<0.05 versus the NCD group, #P<0.05 versus the HFD group. (n=10). All the data is presented as mean±SD (One-way *NOVA*).

Figure S1

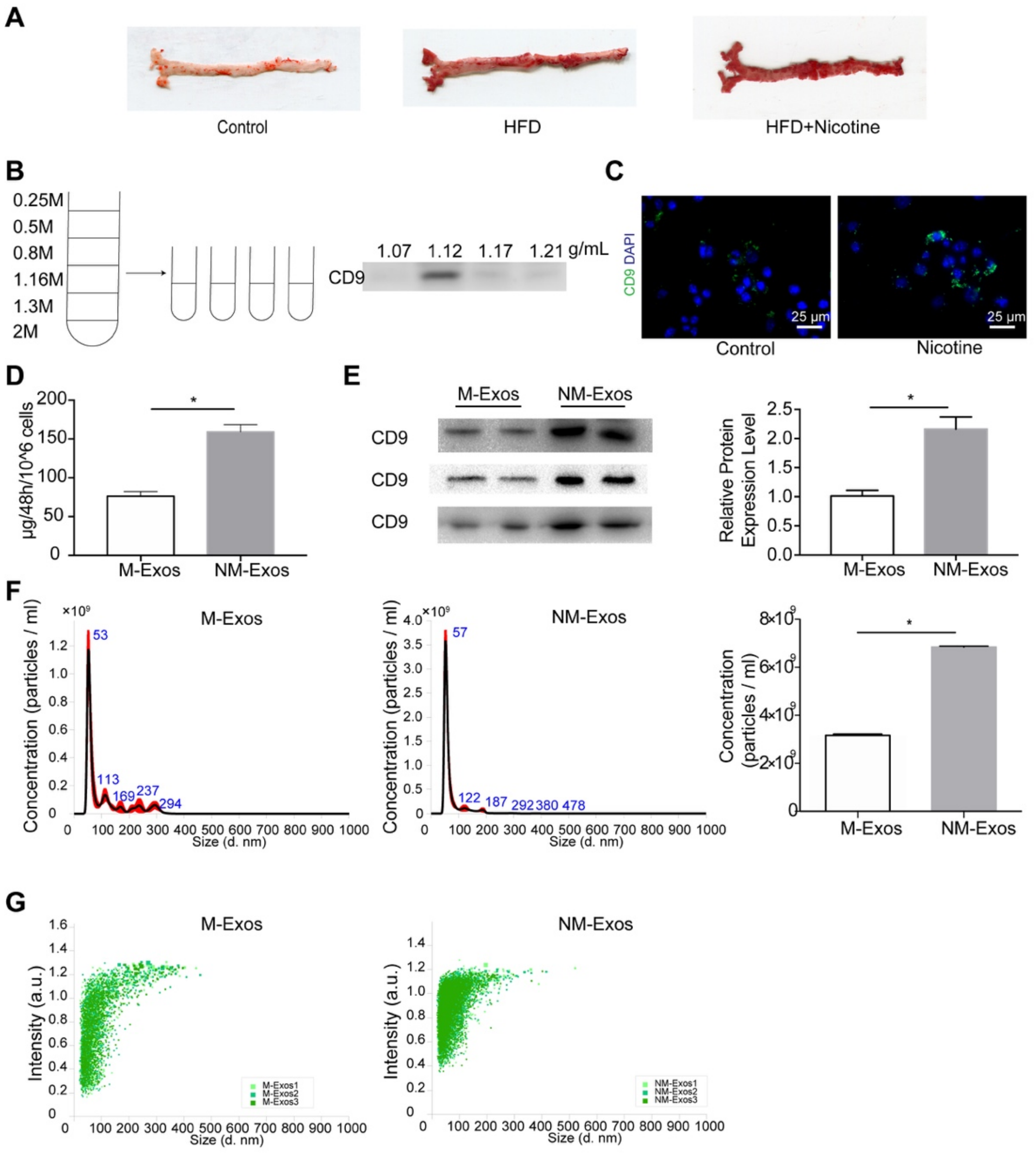

Figure S1. (A) The Oil Red O staing of *ApoE*$^{-/-}$ mice aortas in different groups. (B) Sucrose gradient density centrifugation and western blot of recovered proteins in a sucrose gradient for exosomes markers. (C) The intracellular exosomes marker expression post nicotine administration. Magnification ×630. (D) Capacity of exosomes production post nicotine stimulation (n=5). (E) Western blotting of CD9 for total exosomal protein from the same number of control and nicotine-treated cells (n=3). (F) NTA particle concentration results for M-Exos and NM-Exos from 10^7 cells for 48 h (n=3). (G) NTA particle size distribution in form of intensity for M-Exos and NM-Exos from 10^7 cells for 48 h (n=3). *P<0.05. All the data is presented as mean±SD (*Student's t-test*).

Figure S2

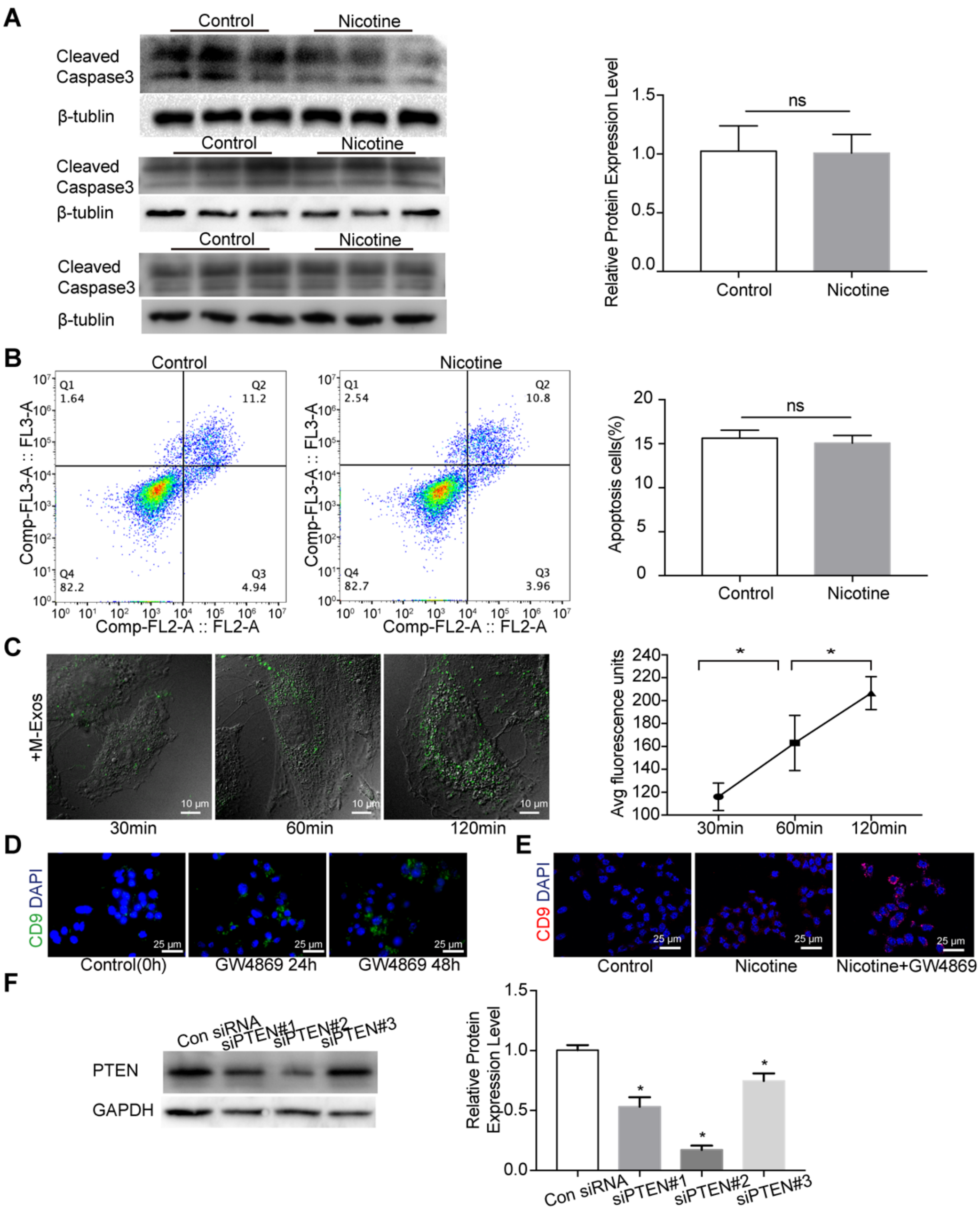

Figure S2. (A) Western blotting of RAW264.7 apoptotic level post nicotine stimulation (n=3). (B) Flow cytometry of RAW264.7 apoptotic level post nicotine stimulation (n=3). (C) Fluorescence images of VSMCs incubated with PKH-67-labelled M-Exos (green) for 30min, 60min and 120min and quantification of internalized exosomes were measured by average fluorescence units per sight (n=5). Magnification ×630. (D) The intracellular exosomes marker expression post GW4869 administration. Magnification ×630. (E) The intracellular exosomes marker expression post nicotine treatment with or without GW4869 administration. Magnification ×630. (F) The western blotting results for PTEN post siPTENs transfection (n=3). ns, not significant; *P<0.05. All the data is presented as mean±SD (*Student's t-test* and One-way *NOVA*).
